# Muscle Cells Provide Instructions for Planarian Regeneration

**DOI:** 10.1016/j.celrep.2013.07.022

**Published:** 2013-08-15

**Authors:** Jessica N. Witchley, Mirjam Mayer, Daniel E. Wagner, Jared H. Owen, Peter W. Reddien

**Affiliations:** 1 Howard Hughes Medical Institute, 4000 Jones Bridge Road, Chevy Chase, MD 20815, USA; 2 Whitehead Institute, 9 Cambridge Center, Cambridge, MA 02142, USA; 3 Department of Biology, Massachusetts Institute of Technology, 9 Cambridge Center, Cambridge, MA 02142, USA

## Abstract

Regeneration requires both potential and instructions for tissue replacement. In planarians, pluripotent stem cells have the potential to produce all new tissue. The identities of the cells that provide regeneration instructions are unknown. Here, we report that position control genes (PCGs) that control regeneration and tissue turnover are expressed in a subepidermal layer of nonneoblast cells. These subepidermal cells coexpress many PCGs. We propose that these subepidermal cells provide a system of body coordinates and positional information for regeneration, and identify them to be muscle cells of the planarian body wall. Almost all planarian muscle cells express PCGs, suggesting a dual function: contraction and control of patterning. PCG expression is dynamic in muscle cells after injury, even in the absence of neoblasts, suggesting that muscle is instructive for regeneration. We conclude that planarian regeneration involves two highly flexible systems: pluripotent neoblasts that can generate any new cell type and muscle cells that provide positional instructions for the regeneration of any body region.

## INTRODUCTION

Cellular models for regeneration must explain two essential attributes of adult tissues: the potential for regeneration and the information to guide regeneration. The potential for regeneration refers to the capacity of particular adult cells to replace missing cells (Tanaka and Reddien, 2011). The information for regeneration refers to the molecular instructions that guide which cell types are regenerated. Positional information for regeneration has long been recognized as a key issue (French et al., 1976; Wolpert, 1969), and therefore it is critical to determine the cellular source of adult positional information. Transplantation experiments and Hox gene-expression analyses in vertebrate skin indicate that dermal fibroblasts can influence epithelial positional identity (Dhouailly, 1984; Rinn et al., 2006, 2008). In amphibian limb regeneration, nerves, connective tissue, and epidermis have all been implicated in affecting patterning during regeneration (Nacu et al., 2013; Nacu and Tanaka, 2011). *Hydra* have a body column comprised of ectodermal and endodermal epithelial cells, which possess muscle- like features (myoepithelial cells). *Wnt* genes are expressed in both epithelial layers near the *Hydra* head, and Wnt signaling promotes *Hydra* head regeneration (Broun et al., 2005; Hobmayer et al., 2000; Lengfeld et al., 2009). Despite these advances, how positional identities are established, maintained, and regenerated in adult tissues is poorly understood.

Planarians are flatworms and constitute a classic regeneration model system (Reddien and Sánchez Alvarado, 2004). They can regenerate any missing body part and maintain adult tissues by replacing aging differentiated cells. New cells in planarian regeneration and tissue turnover are produced by neoblasts, adult proliferative cells that include pluripotent stem cells (cNeoblasts) (Reddien and Sánchez Alvarado, 2004; Wagner et al., 2011). The neoblast population therefore harbors the potential for regeneration and tissue turnover. However, it is unknown which cells possess positional information for planarian regeneration. Transplantation of tissues from one body region to another can trigger intercalary regeneration in many regenerative organisms (French et al., 1976; Reddien and Sánchez Alvarado, 2004; Santos, 1931). In intercalary regeneration, missing positional coordinates can be regenerated between juxtaposed tissues, sometimes leading to outgrowths. For example, a cylindrical plug of planarian tissue that has been flipped and inserted (with an inverted dorsoventral [DV] axis) triggers outgrowths (Okada and Sugino, 1937). Irradiation eliminates neoblasts (Dubois, 1949), and yet irradiated DV-inverted plugs still trigger outgrowths in unirradiated hosts, suggesting that positional information might exist in differentiated planarian tissues (Kato et al., 2001).

The molecular basis for positional information (i.e., genes controlling pattern formation) as a biological problem has been investigated primarily in animal embryos and remains understudied in adult tissues. Molecular genetic studies in planarians have revealed that orthologs of numerous embryonic patterning genes in other organisms have roles in adult planarian tissues for instructing tissue turnover and regeneration (Reddien, 2011). In this study, we define position control genes (PCGs) as genes that (1) display regionalized expression along one or more body axes, and (2) either show a patterning-abnormal RNAi phenotype (e.g., homeotic) or encode a protein that is predicted to regulate pathways (e.g., Wnt, Bmp, or Fgf signaling) that are important for planarian patterning (Reddien, 2011). Most PCGs encode signaling pathway receptors, ligands, or secreted inhibitors. We analyzed more than 20 genes that met these criteria and had expression domains spanning different regions of all body axes. Several examples illustrate PCG properties: *wnt1* is expressed at the animal tail tip and at all wounds, and *wnt1* RNAi causes regeneration of heads in place of tails (Adell et al., 2009; Petersen and Reddien, 2009b), a phenotype that is also observed following RNAi of the Wnt pathway b*-catenin-1* gene (Gurley et al., 2008; Iglesias et al., 2008; Petersen and Reddien, 2008). *notum* is expressed at the anterior pole and anterior-facing wound sites, and *notum* RNAi causes regeneration of tails in place of heads (Petersen and Reddien, 2011). *bmp4* is expressed in a medial-to-lateral messenger RNA (mRNA) gradient on the dorsal side (Orii et al., 1998), and RNAi of *bmp4* causes ventralization (Molina et al., 2007; Orii and Watanabe, 2007; Reddien et al., 2007). In all of these cases, the site of PCG expression corresponds to the body region that is affected by RNAi of that gene. Here, we address the cellular source of the positional information for planarian regeneration by investigating planarian PCGs.

## RESULTS

### Positional Information Exists in Differentiated Planarian Tissues

**Figure 1:**
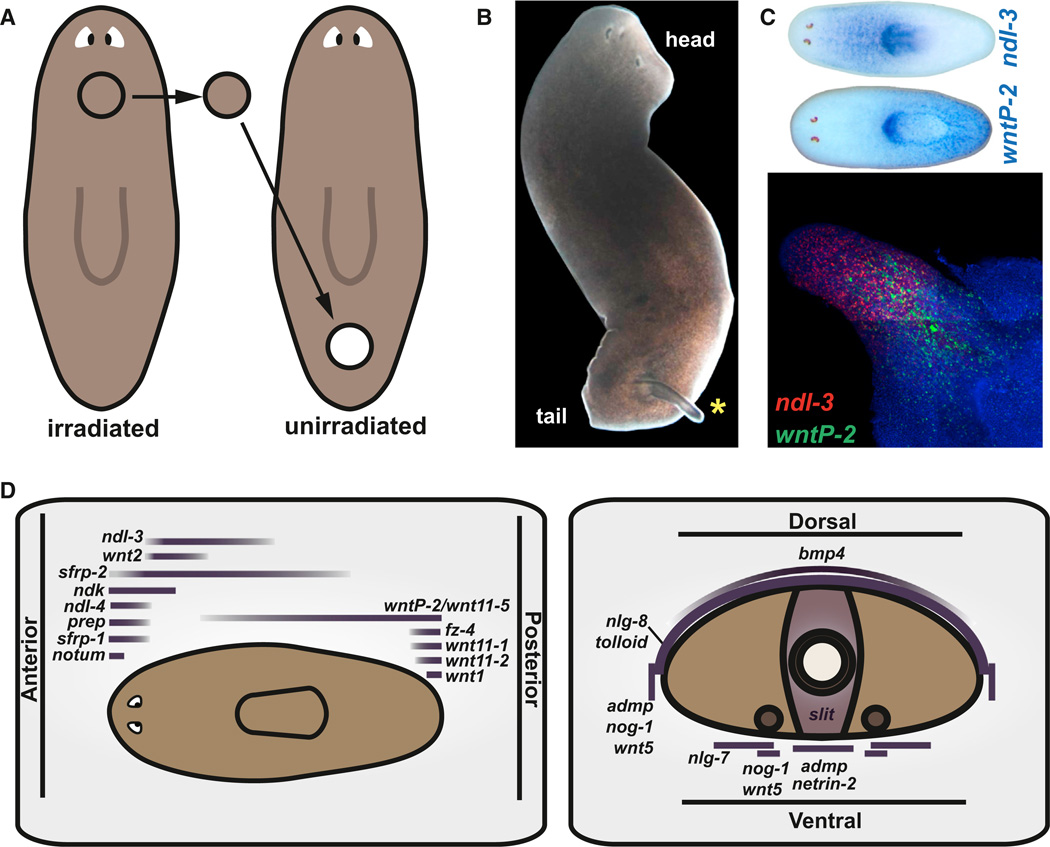
Positional Conflict without Neoblasts (A) Anterior cylindrical plugs from animals irradiated with 6,000 rad were transplanted into the posterior of unirradiated hosts. For other transplant experiments, see Kato et al., 2001; Saló and Baguñà, 1985. (B) Tissue outgrowth was triggered by transplantation (yellow asterisk). (C) *ndl-3* and *wntP-2* were respectively expressed in the anterior and posterior of wild-type animals, and in the distal and proximal regions of an outgrowth. (D) Cartoon depicting domains of PCG expression, which define positions across AP, DV, and ML body axes (modified from Reddien, 2011). See also Figure S1.

A previous study by (Saló and Baguñà, 1985) revealed that tissue regeneration can occur between unirradiated grafts from one anteroposterior (AP) region transplanted into irradiated hosts at a different AP region. In that study, surgical trimming resulted in a wounded, transplanted fragment representing one AP end of the animal (Saló and Baguñà, 1985), and in principle, this wounded fragment end could have influenced the regenerative response. Nonetheless, in this work, we found that cylindrical plugs from the anterior of irradiated animals triggered outgrowth upon transplantation into the posterior of otherwise intact, unirradiated animals (Figures 1A and 1B). The distal outgrowth end expressed an anterior PCG and the proximal end a posterior PCG (Figure 1C). These observations, together with results from additional transplantation experiments (Kato et al., 2001; Saló and Baguñà, 1985), suggest that differentiated planarian cells (nonneoblasts) have positional information that can instruct regenerative responses.

**Figure 2:**
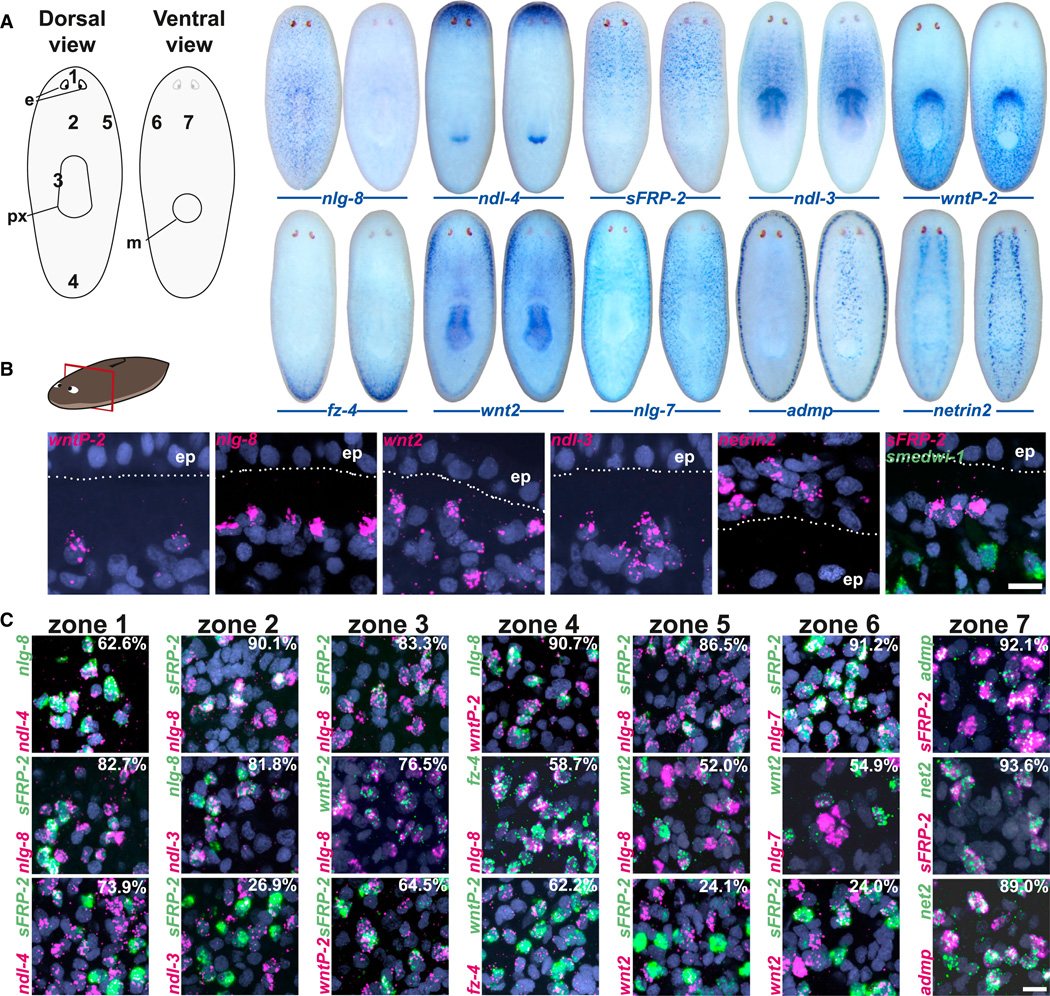
PCGs Are Coexpressed in Subepidermal Cells (A) Expression patterns of the PCGs analyzed in this figure (see also Figure S1). Cartoon depicts seven analyzed zones (zones 1–7). Anterior: up; left animal: dorsal view; right animal: ventral view. Animals are 1.5–2.5 mm in length. e, eye; m, mouth; px, pharynx. (B) Fluorescent images of transverse tissue sections. Dorsal up, as illustrated. ep, epidermis. (C) Three RNA probe pairs were used in double FISH experiments. The percentage of cells expressing the less-abundant (fewer cells) gene that also expressed the more-abundant gene is shown (the less-abundant gene is green in each pair). Three animals were counted and the data were summed for each gene pair. Nuclear signal (DAPI) is shown in blue. Scale bar, 10 µm (B and C). See also Figures S2 and S3.

PCG expression patterns define unique domains along the AP, DV, and mediolateral (ML) axes (Reddien, 2011; Figure 1D). Several PCGs have patterning abnormal RNAi phenotypes (Table S1). Other PCGs are homologous to patterning pathway regulators, but do not yet have reported RNAi phenotypes that define their regeneration roles (Table S1). All PCGs examined (Figures 1D, 2A, and S1A) displayed a similar feature: expression in a peripheral, subepidermal cell layer (Figures 2B and S1B; Gaviño and Reddien, 2011; Petersen and Reddien, 2009b, 2011; Reddien et al., 2007) that is devoid of neoblasts (Figures 2B and S1B). Furthermore, irradiation rapidly eliminated neoblasts, but not PCG expression (Figures S2A and S2B; Gaviño and Reddien, 2011; Gurley et al., 2010; Petersen and Reddien, 2009b, 2011; Reddien et al., 2007). These observations indicate that the major site of PCG expression is in nonneoblast cells.

The common subepidermal location of PCG expression raised the possibility that a specific subepidermal cell population plays an informational role in tissue turnover and regeneration. We refer to this candidate cell population as “position control cells” (PCCs), and hypothesize that this PCC population harbors positional information, thus providing body coordinates for maintaining the adult body plan during tissue turnover and guiding regeneration (a biological GPS-like system, with numerous static cell satellites). If this is correct, PCCs should have a broad distribution (to provide information across all body axes, and because regeneration can start from essentially any body fragment), they should be capable of simultaneously expressing multiple patterning factors to define a coordinate system, should dynamically adjust expression during regeneration to produce a new coordinate system, and should not depend on neoblasts for maintenance of PCG expression or for some changes in pattern of expression after injury.

### PCGs Are Expressed in the Same Cell Population

To determine whether a PCC population exists, we first asked whether PCGs are expressed together in the same cells. We analyzed three separate PCG pairs by double fluorescence in situ hybridization (FISH) in each of seven zones, which spanned the AP, DV, and ML axes (Figures 2C and S3A). In every case examined, PCGs were coexpressed in the same cells to a substantial degree. For example, 82.7% of *sFRP-2*^+^ (anterior marker) cells were also *nlg-8*^+^ (dorsal marker) in the dorsal-head region (zone 1; Figure 2C). Similarly, 73.9% of *sFRP-2*^+^ zone 1 cells were *ndl-4*^+^, and 62.6% of *nlg-8*^+^ zone 1 cells were *ndl-4*^+^. Similar results were obtained in all seven investigated zones, with coexpression percentages ranging from 24% to 93.6% (Figure 2C). At all analyzed locations, cells coexpressed regulators of multiple signaling pathways and both AP and DV PCGs. These data are consistent with the possible existence of a PCC population.

### Planarian Muscle as a Positional Coordinate System

**Figure 3:**
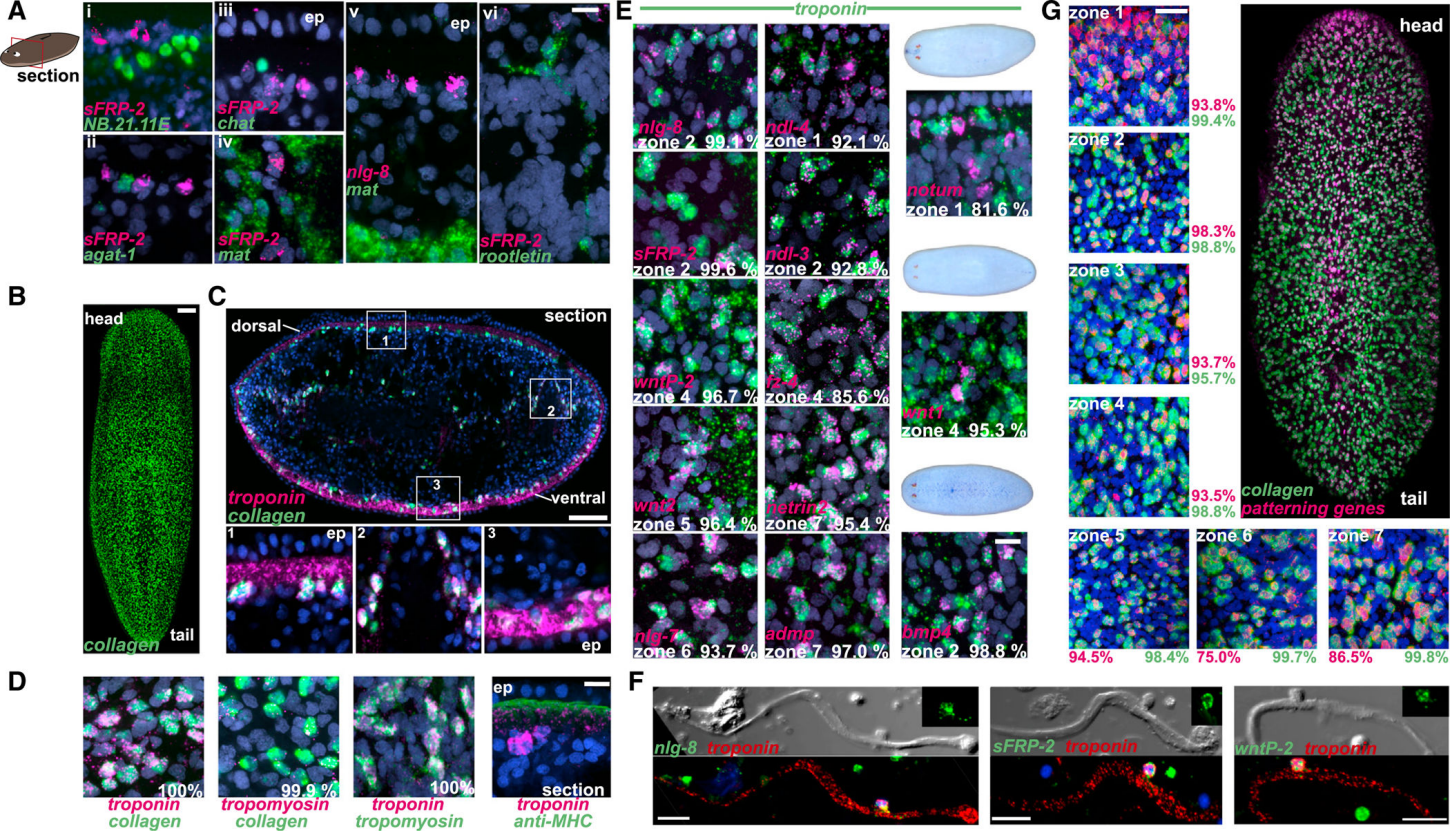
PCGs Are Expressed in Muscle Cells (A) Prepharyngeal sections (dorsal up; compare with the cartoon) show that position control cells (*sFRP-2*^+^ or *nlg-8*^+^) are distinct from specific peripheral neoblast progeny cell types (i, *NB.21.11e*^+^; ii, *agat-1*^+^), peripheral neurons (iii, *chat*^+^), intestinal cells (iv and v, *mat*^+^), and protonephridia (vi, *rootletin*^+^). Internal *sFRP-2*^+^ cells close to intestine (iv) and *nlg-8*^+^ cells far from intestine (v) are shown. ep, epidermis. (B) *collagen* is broadly expressed subepidermally, similar to the density and location of position control cells (dorsal view shown; anterior up). (C) *collagen*^+^ cells are muscle cells that coexpress *troponin* both subepidermally (insets 1 and 3) and internally (inset 2). (D) *collagen*^+^ cells coexpress both *tropomyosin and troponin*. The percentage of *collagen*^+^ cells (>500 cells counted from three animals each) expressing *troponin/tropomyosin* is shown. *troponin* and *tropomyosin* are also coexpressed in the same cells. Right: cross-section showing subepidermal *troponin*^+^ cells with signal extending into the region of muscle fibers (labeled with the anti-myosin-heavy-chain (MHC) antibody TMUS-13; Cebrià and Vispo, 1997). (E) PCGs are coexpressed with *troponin* (the percentage of position control cells expressing *troponin* is shown; >250 position control cells were examined from three animals each). Axis regeneration genes *notum* (anterior regeneration), *wnt1* (posterior regeneration), and *bmp4* (dorsal regeneration) were also coexpressed with *troponin* (115, 41, and 327 cells, respectively, were examined). Additional examples are provided in Figure S3B. (F) Differential interference contrast (DIC, top) and fluorescent micrograph (bottom) of isolated muscle fibers expressing *troponin* and PCGs (six *sFRP-2*^+^, four *nlg8*^+^, and four *wntP-2*^+^ cells were examined). Nuclei (DAPI) are blue. Additional green signal is in debris (lacking a nucleus). (G) Coexpression of 19 PCGs (red, combined RNA probes) and *collagen* (green) in zones 1–7 (as in Figure 2). The percentage of double-positive cells is given as a fraction of all PCG-positive cells (red) and all *collagen*^+^ cells (green; three animals examined for each region). Neural *netrin1* and *netrin2* expression affects the percentage of red cells that are *collagen*^+^ in zones 6 and 7. Scale bars, 10 µm (A, C [bottom], and D–F), 100 µm (B and C [top]), 200 µm (E), and 20 µm (G). See also Figure S1.

To determine the identity of cells expressing PCGs, we analyzed the relation of cells expressing PCGs *sFRP-2* and *nlg-8* to various known differentiated cell types. *sFRP-2* was not detectably coexpressed in subepidermal neoblast progeny populations (*NB.21.11E*^+^ and *agat-1*^+^; Eisenhoffer et al., 2008), peripheral neurons (*chat*^+^), intestinal cells (*mat*^+^), or protonephridial tubules (*rootletin*^+^; Figure 3A). We did, however, identify a cell population with a relatively homogeneous subepidermal distribution resembling putative PCCs, marked by the expression of *collagen* (Figure 3B). We next determined these *collagen*^+^ cells to be muscle cells, coexpressing muscle markers *troponin* and *tropomyosin* (Figures 3C and 3D). Topologically, planarian muscle nuclei are internal to four layers of muscle fibers, and we found that *collagen, tropomyosin*, and *troponin* mRNA were concentrated around muscle nuclei, with *troponin* and *tropomyosin* mRNA extending into muscle fibers (Figures 3C and 3D; Movies S1 and S2). The distribution of muscle cells is consistent with the possibility that these cells are PCCs. We therefore tested for coexpression of PCGs and muscle markers.

Every PCG analyzed, including *nlg-8, ndl-4, sFRP-2, ndl-3, wntP-2* (also known as *wnt11-5*), *fz-4, wnt2, nlg-7, admp, netrin2, notum, wnt1, bmp4, wntA, wnt5, ndk, tolloid, wnt11-1, wnt11-2, sFRP-1*, and ne*trin1*, was coexpressed with *troponin* or *collagen* (Figures 3E, S3B, and S3C). The percentage overlap in expression of most PCGs and muscle markers was remarkably high. For example, 99.1% of *nlg-8*^+^ cells, 96.7% of *wntP-2*^+^ cells, and 98.8% of *bmp4*^+^ cells were *troponin*^+^. PCG mRNA was localized around the nucleus of *troponin*^+^ cells, possibly because most of these genes encode transmembrane or secreted proteins, which might be localized with perinuclear endoplasmic reticulum-associated ribosomes.

To further demonstrate that cells expressing PCGs are muscle cells, we performed FISH on isolated muscle fibers. We developed an enzymatic tissue-dissociation procedure capable of isolating intact, contractile muscle fibers (Movie S3) with associated offset nuclei (Figure 3F), a known planarian muscle cell morphology (Gelei, 1927). *sFRP-2, wntP-2*, and *nlg-8* mRNAs were present around the nuclei of isolated *troponin*^+^ muscle cells, with some signal also observed in the fiber proximal to the nucleus (Figure 3F). We conclude that PCGs are expressed in muscle cells of unamputated planarians. The PCG expression patterns in muscle described here explain the reported expression patterns for almost every known planarian PCG discovered from extensive RNAi and gene-expression studies over the past 10 years (Adell et al., 2009; Cebrià et al., 2002; Cebrià and Newmark, 2005; Gaviño and Reddien, 2011; Gurley et al., 2008, 2010; Iglesias et al., 2008; Kobayashi et al., 2007; Molina et al., 2007, 2009, 2011; Orii and Watanabe, 2007; Petersen and Reddien, 2008, 2009b, 2011; Reddien et al., 2005a, 2007; Rink et al., 2009). These findings suggest that muscle is a major site of instructive signaling in planarian regeneration.

To determine whether a minority or majority of muscle cells expressed PCGs, we simultaneously hybridized RNA probes for 19 PCGs and detected the combined signal in the red channel, while detecting *collagen* signal in the green channel. Between 95.7% and 99.8% of muscle cells analyzed in the seven regions described above displayed signal from the combined PCGs (Figures 3G, S3D, and S3E). Therefore, in addition to contraction, expression of PCGs is a major attribute of muscle throughout the planarian body wall.

Most PCGs displayed little to no detectable expression outside of subepidermal muscle cells (Figures 3G and S1), but some PCGs were expressed internally. For example, *nlg-8* and *sFRP-2* were expressed in intestinal muscle cells (Figure S3F), several PCGs were expressed in pharyngeal muscle cells (Figure S3G), and *wntP-2* was strongly expressed in muscle cells anterior to the pharynx (Figure S3H). These analyses further demonstrate the capacity of muscle cells to express PCGs, even at locations outside the body wall. Finally, some PCGs also displayed expression in *troponin*^−^ cells in the brain (*wnt1* and *wntA*, Figure S3I), nerve cords or other neurons (*netrin2*), or in a small ring at the anterior end of the pharynx (*bmp4*; Figure S3J), indicating potential additional functions at other locations for these genes.

### Dynamic Change in PCG Expression in Muscle during Regeneration

**Figure 4:**
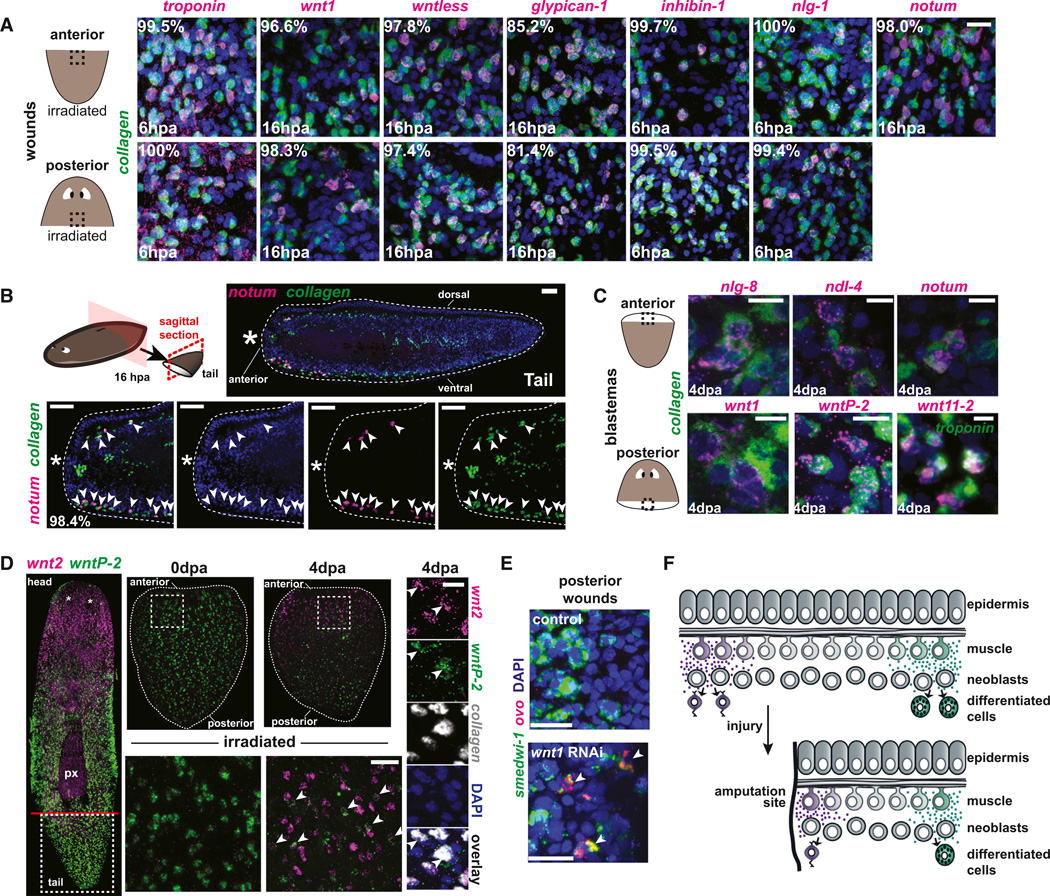
Dynamic PCG Expression in Muscle Cells following Amputation (A) Animals were irradiated with 6,000 rads (eliminating neoblasts) and amputated transversely 3 days later. The percentage of PCG^+^ cells at wounds expressing *collagen* or *troponin* was examined in three animals each. Anterior-facing wounds are from tails, and posterior-facing wounds are from heads (except for *nlg-1* and *glypican*, where one anterior-facing wound was from a trunk; 117–547 cells were examined for each condition); 108/108 and 423/425 *collagen*^+^ cells were *troponin*^+^ at anterior- and posterior-facing wounds, respectively, at 6 hours postamputation (6 hpa; 168/168 and 141/141 were double positive at 16 hpa). (B) Schematic illustrating the surgical procedure. Tail fragments were fixed at 16 hpa, labeled by FISH and DAPI, and analyzed in sagittal sections. Anterior is left. Bottom: zoomed images from the anterior-facing wound site. Arrowheads indicate coexpression of *notum* and *collagen* in cells adjacent to the amputation plane (n = 418/425 were double positive). (C) PCGs with regeneration RNAi phenotypes are coexpressed with *collagen* or *troponin* in blastemas (the time after amputation is indicated). (D) Transient coexpression of anterior-specific (*wnt2*) and posterior-specific (*wntP-2*) PCGs during regeneration. Animals were irradiated (6,000 rads) and amputated 3 days later. Left: intact animal (3 days postirradiation). Center: tail fragments (bottom, zoomed) at 0 dpa and 4 dpa. *wnt2* was coexpressed with *wntP-2* at 4 dpa. Right: Triple-color FISH demonstrates coexpression of *wnt2, wntP-2*, and *collagen*. (E) *wnt1(RNAi)* animals had clusters of numerous *ovo*^+^ cells at posterior-facing wounds at 4 dpa (n = 2/5 animals, with three total clusters present and none present in control RNAi [n = 4] animals; see also Figure S4E). All posterior *ovo*^+^ cell clusters in *wnt1(RNAi)* animals possessed multiple *smedwi-1*^+^/*ovo*^+^ cells (arrowheads; *smedwi-1* expression marks neoblasts). (F) Model. PCG expression in muscle specifies the identity of new cell types made in tissue turnover. Following amputation, muscle cells change their PCG expression, and these changes dictate which type of new tissue is regenerated. The influence of muscle on neoblasts is depicted by purple and green signaling environments, but it need not be direct. Scale bars, 20 µm (A), 50 µm (B), and 10 µm (C and D). See also Figure S1.

The concept of GPS-like PCCs could explain the maintenance of regional tissue identities during cell turnover, and the capacity to regenerate missing tissues if PCCs can alter their PCG expression following injury. We therefore examined PCG expression during regeneration. Among numerous known wound-induced genes (Wenemoser et al., 2012) is a class (termed W2) that is induced proximal to wounds and includes several PCGs (e.g., *wnt1, wntless, glypican-1*, and *notum*) (Adell et al., 2009; Petersen and Reddien, 2009b; Wenemoser et al., 2012). *wnt1, wntless*, and *notum* RNAi perturb the head-versus-tail regeneration choice at transverse wounds (Adell et al., 2009; Petersen and Reddien, 2009b, 2011), and *glypican-1* RNAi causes indented head regeneration (Wenemoser et al., 2012), a phenotype similar to that of Bmp-defective planarians. These wound-induced genes (*wnt1, wntless, glypican-1*, and *notum*), the additional wound-induced genes *inhibin-1* and *nlg-1*, and *bmp4* in parasagittal fragments were all expressed in muscle cells at wounds (Figures 4A, S4A, and S4B). The percentage overlap of expression of W2 genes and muscle markers at wounds was very high. For example, 96.6% of *wnt1*^+^ cells, 99.7% of *inhibin*^+^ cells, 100% of *nlg-1*^+^ cells, and 98% of *notum*^+^ cells were detectably *collagen*^+^ at anteriorfacing wounds. Tissue sections of wounded animals at multiple body locations showed clear subepidermal, wound-induced expression of *notum* and *wnt1* restricted to *collagen*^+^ cells (98.4% and 95.7%, respectively, were detectably double positive; Figures 4B, S4C, and S4D). Furthermore, wound-induced expression of the PCGs in muscle cells occurred in irradiated animals, indicating that existing muscle cells at wounds can change their PCG expression profile. Finally, similar to the case for wound-induced PCG expression, cells expressing PCGs in head and tail blastemas were also *troponin*^+^ (Figure 4C).

We tested whether muscle cells can dynamically adopt new positional identities in response to amputation. Upon amputation, positional information in body fragments must be read-justed to regenerate an appropriately proportioned animal. For example, following transection, tail fragments express anterior-specific PCGs at their anterior end, whereas posterior-specific PCG expression can become posteriorly restricted to the tail fragment tip (Gurley et al., 2010; Petersen and Reddien, 2009a). Some of these expression changes specific to anterior or posterior regeneration also occur in the absence of neoblasts (Gurley et al., 2010; Petersen and Reddien, 2009a). We examined expression of *wnt2*, which is expressed in the head, and *wntP-2*, which is expressed in a broad posterior-to-anterior gradient emanating from the tail tip (Figures 1D and S1), utilizing amputated tail fragments that had been previously irradiated to prevent new cell formation. Following amputation, *wnt2* was expressed in anterior muscle cells of the tail fragment and *wntP-2* expression receded to muscle cells of the tail tip (Figure 4D). The expression domains of these genes were thus partially regenerated in the existing muscle cells of the irradiated tail fragments, which were incapable of new tissue regeneration. A considerable fraction of anterior *collagen*^+^ muscle cells showed both new *wnt2* expression and retained *wntP-2* expression (45.6% at 2 days and 66.4% at 4 days; Figure 4D). Coexpression of *wnt2* and *wntP-2* further indicated that previously posterior muscle cells can express anterior PCGs while simultaneously decreasing posterior PCGs after amputation.

Finally, we assessed neoblast lineage specification following RNAi of genes that are expressed in muscle at wound sites: *wnt1* and *notum. smedwi-1* is expressed specifically in neoblasts (Reddien et al., 2005b) and *ovo* is expressed in a subset of *smedwi-1*^+^ neoblasts at anterior-facing wounds that are progenitors for eye regeneration (Lapan and Reddien, 2012). *ovo^+^/smedwi*^+^ cells were present at posterior-facing wounds following *wnt1* RNAi, but not in the control (4 days postamputation [4 dpa]; Figures 4E and S4E). Conversely, smaller than normal numbers of *ovo*^+^ cells, including *ovo^+^/smedwi*^+^ cells, were present at anterior-facing wounds in *notum(RNAi)* animals (Figure S4F). These data demonstrate that the fates of at least some neoblasts are dependent on *wnt1* and *notum*, which were induced in muscle at wounds (Figures 4A, 4B, S4A, and S4C). Whether this reflects direct action on neoblasts by WNT1/NOTUM proteins is unknown. Because PCGs can be instructive for regeneration (e.g., *wnt1* and *notum*) and some PCG expression domains can be regenerated in existing muscle tissue in irradiated amputated animals, we propose that changes in muscular PCG expression functions instructively for downstream regeneration programs (Figure 4F).

## DISCUSSION

Positional information is essential for maintaining regional tissue identity during tissue turnover and wound repair in most adult animals, but it remains poorly understood. Planarians represent an ideal system for molecular and cellular investigations of adult positional information because of their extensive tissue turnover and regeneration, and because planarian gene function can readily be studied in adults via RNAi and in situ hybridizations. In principle, adult tissue identity could be retained during tissue turnover by cell populations that autonomously maintain their regional identity (e.g., stem cells). By contrast, genes that are expressed regionally in differentiated adult cells could regulate one another through signaling pathways such that a stable pattern is maintained and influences the fate of resident stem cells. Our data from planarians favor the latter model: patterns of PCG expression exist in adult muscle cells, but not in neoblasts, and these patterns are maintained in adult life and facilitate scalability during growth. Besides muscle, other cell types likely have roles in planarian regeneration. Which cells and signals regulate neoblast maintenance, proliferation, migration to wounds, and specialization remains largely unknown. Regardless, our results demonstrate that almost all of the planarian PCG expression patterns previously reported—for genes with roles ranging from maintaining the DV axis (*bmp*) to mediating the head-tail regeneration choice (*wnt1* and *notum*)—are localized in muscle, indicating that subepidermal planarian muscle tissue is a major source of the positional information that guides tissue turnover and regeneration programs.

Injuries remove local patterning instructions, requiring the pattern of PCG expression in muscle itself to be regenerated. We propose a model in which expression changes in existing muscle cells occur as an early and instructive process in regeneration, with positional information being dynamic in an initially static set of muscle cells (Figure 4F). In this model, new PCG expression in muscle at wounds influences (directly or indirectly) the cell types made by neoblasts, promoting regeneration of missing cell types (Figure 4F). How the PCG pattern in muscle is reestablished after injury is largely unknown, but events involved in the head-versus-tail regeneration choice (regeneration polarity) at transverse wounds are the best understood (Gurley et al., 2008, 2010; Iglesias et al., 2008; Petersen and Reddien, 2008, 2009b, 2011). The previously described mechanism for regeneration polarity can be viewed together with the findings reported here in a model for AP axis regeneration: wound signaling generically activates *wnt1* expression in muscle cells at wounds, giving any wound the chance to regenerate a tail if no posterior tissue juxtaposes the wound. At anterior-facing wounds, *notum* is activated in muscle cells and inhibits Wnt signaling to give any anterior-facing wound the opportunity to regenerate a head if it is not juxtaposed by anterior tissue. Initiation of head or tail regeneration is followed by pattern restoration involving the rescaling of existing PCG gradients in muscle (e.g., *wntP-2/wnt11-5* in tail fragments) and the emergence of missing patterns of gene expression, likewise in muscle (e.g., *wnt2* in tail fragments). DV and ML patterns must also be regenerated, and the genes involved in determining these axes also show dynamic expression in muscle. We propose that a combination of two flexible cell types—muscle cells that are capable of expressing position control genes for any body region, and cNeoblasts that are capable of generating all differentiated cell types—enables the dramatic ability of planarians to regenerate any missing body part.

## EXPERIMENTAL PROCEDURES

### Fixations, In Situ Hybridizations, and Immunostainings

Nitroblue tetrazolium/5-bromo-4-chloro-3-indolyl phosphate (NBT/BCIP) colorimetric in situ hybridization and FISH were performed as previously described (Pearson et al., 2009). Animals were placed in 5% N-acetyl-cysteine in 13 PBS for 5 min at room temperature followed by fixation in 4% formaldehyde. Animals were bleached in 6% hydrogen peroxide overnight and stored in methanol at −20°C. Digoxigenin (DIG)-, fluorescein-, and dinitrophenol (DNP)-labeled riboprobes were synthesized as previously described (Pearson et al., 2009). For double and triple labeling, horseradish peroxidase (HRP) inactivation was performed for 45 min between labelings in 4% formaldehyde diluted in PBSTx (0.1% Triton X-100). Some HRP inactivations were performed overnight (≥16 hr) in1% w/v (154 mM) Na Azide dissolved in PBSTx. To detect the expression of multiple PCGs at the same time in the same channel (Figure 3G), DIG probes against 19 GPS genes (*ndk, ndl-4, ndl-3, notum, sFRP-1, sFRP-2, wnt2, wntA, wnt11-1, wnt11-2, wntP-2, wnt1, wnt5, bmp4, nlg-7, slit, admp, netrin1*, and *netrin2*) were hybridized simultaneously, together with a DNP probe against *collagen*. After mRNA labeling was completed, samples were blocked for immunolabeling when used in PBSTB (0.3% Triton X-100, 0.25% BSA) and incubated overnight at room temperature in the monoclonal antibody TMUS13 (kind gift of Dr. Rafael Romero) diluted 1:10 in PBSTB. Linked primary TMUS13 antibody was detected with a secondary Alexa488-coupled anti-mouse antibody (1:650 in PBSTB). Gene model sequences for *collagen* (SMED_00066_V2), *troponin* (SMED_00109_V2), and *tropomyosin* (SMED_00440_V2) are available at GEO (GPL14150, see Supplementary File GPL14150_gene_models.txt).

### Microscopy and Image Analysis

Fluorescent micrographs were acquired using the Zen software on a Zeiss LSM 700 laser scanning confocal microscope and 405 nm, 488 nm, 555 nm, and 639 nm laser lines. Image analysis was performed using ImageJ and Fiji. To determine the percentage of coexpression of multiple PCGs, cells were quantified in triplicate from stacks within defined regions of each of seven zones. Cells were counted using the Cell Counter plugin (http://rsbweb.nih.gov/ij/plugins/cell-counter.html).

### Enzymatic Preparation of Individual Muscle Fibers

Whole worms were amputated into sagittal strips in CMF (15 mM HEPES, 400 mg/l NaH_2_PO_4_, 800 mg/l NaCl, 1,200 mg/l KCl, 800 mg/l NaHCO_3_, 240 mg/l glucose, 1% BSA, pH 7.3). Fragments were placed in 100 µl of CMF together with a 1:10 dilution of Liberase TH (Roche), an enzymatic mix of collagenase I and II, and a neutral protease, and incubated at 30°C. Mechanical tissue loosening involved gentle tube flicking and slow pipetting with a cut pipet tip approximately every 15–20 min. Too vigorous mechanical manipulation resulted in shearing of fibers; mixing frequency was determined based upon tissue appearance using Nomarski optics during dissociation. Tissues were incubated in liberase in CMF for 45 min to 1.5 hr, depending on the starting fragment size and handling, until suitable dissociation occurred.

### RNAi

Animals were injected with *notum* and *wnt1* double-stranded RNA (dsRNA) after head and tail amputation, injected again the next day, and then left to rest for 1 day. This 3-day procedure was repeated two consecutive times, and the animals were fixed 48 hr or 4 days after the final amputation. *Caenorhabditis elegans unc-22* dsRNA served as the RNAi control.

## Supplementary Material

01

02

03

04

05
