# Supplementary material for: Muscle Cells Provide Instructions for Planarian Regeneration
Source: Cell Rep. Author manuscript; Available in PMC 2014 Jul 17. (PMC4101538; doi:10.1016/j.celrep.2013.07.022)
Supplement: 01 [file NIHMS523206-supplement-01.pdf]

Supplemental Table 1. Position control genes used in manuscript

	**signaling pathway**	**pattern/expression reference**	**patterning RNAi phenotype**	**phenotype reference**
notum	WNT	Petersen 2011	two tails	Petersen 2011
sFRP-1	WNT	Petersen 2008, Gurley 2008		
sFRP-2	WNT	Gurley 2010		
ndl-4	FGF	Rink 2009		
ndl-3	FGF	Rink 2009		
ndk	FGF	Cebria 2002	brain/eye expansion	Cebria 2002
wnt2	WNT	Petersen 2008		
wntA	WNT	Kobayashi 2007, Adell 2009	brain/eye expansion	Kobayashi 2007, Adell 2009
wntP-2	WNT	Petersen 2008, Petersen 2009, Gurley 2010	synergistic with *wnt1*	Petersen 2009
wnt11-1	WNT	Petersen 2008, Gurley 2010		
wnt11-2	WNT	Adell 2009, Gurley 2010	tailless, posterior midline defects	Adell 2009, Gurley 2010
fz-4	WNT	Gurley 2008		
wnt1	WNT	Petersen 2008	two heads	Adell 2009, Petersen 2009
wntless	WNT	Adell 2009	two heads	Adell 2009
bmp4	BMP	Orii 1998, Molina 2007, Reddien 2007, Orii 2007	ventralization	Molina 2007, Reddien 2007, Orii 2007
nlg-8	BMP	Molina 2009	neural ventralization	Molina 2011
nlg-7	BMP	Molina 2009		
tolloid	BMP	Reddien 2007	midline defects	Reddien 2007
admp	BMP	Molina 2011, Gaviño 2011	synergistic with *bmp4*	Gaviño 2011, Molina 2011
nog-1	BMP	Molina 2007, Molina 2009	dorsalization	Molina 2011
glypican-1	BMP	Wenemoser 2012	indented heads, midline defects	Wenemoser 2012
netrin1	netrin/dcc	Cebria 2005		
netrin2	netrin/dcc	Cebria 2005	CNS architecture defects	Cebria 2005
wnt5	WNT	Marsal 2003, Adell 2009	deflected brain, ectopic pharynxes	Adell 2009, Gurley 2010
slit	slit/robo	Cebria 2007	midline defects	Cebria 2007
