# Supplementary material for: Muscle Cells Provide Instructions for Planarian Regeneration
Source: Cell Rep. Author manuscript; Available in PMC 2014 Jul 17. (PMC4101538; doi:10.1016/j.celrep.2013.07.022)
Supplement: 05 [file NIHMS523206-supplement-05.pdf]

Cell Reports
Report

Cell PRESS
Open ACCESS

Muscle Cells Provide Instructions for Planarian Regeneration

Jessica N. Witchley,[1,2,3,4] Mirjam Mayer,[1,2,3,4] Daniel E. Wagner,[1,2,3] Jared H. Owen,[1,2,3] and Peter W. Reddien[1,2,3,*]
[1]Howard Hughes Medical Institute, 4000 Jones Bridge Road, Chevy Chase, MD 20815, USA
[2]Whitehead Institute, 9 Cambridge Center, Cambridge, MA 02142, USA
[3]Department of Biology, Massachusetts Institute of Technology, 9 Cambridge Center, Cambridge, MA 02142, USA
[4]These authors contributed equally to this work
*Correspondence: reddien@wi.mit.edu
http://dx.doi.org/10.1016/j.celrep.2013.07.022
This is an open-access article distributed under the terms of the Creative Commons Attribution License, which permits unrestricted use, distribution, and reproduction in any medium, provided the original author and source are credited.

SUMMARY

Regeneration requires both potential and instructions for tissue replacement. In planarians, pluripotent stem cells have the potential to produce all new tissue. The identities of the cells that provide regeneration instructions are unknown. Here, we report that position control genes (PCGs) that control regeneration and tissue turnover are expressed in a subepidermal layer of nonneoblast cells. These subepidermal cells coexpress many PCGs. We propose that these subepidermal cells provide a system of body coordinates and positional information for regeneration, and identify them to be muscle cells of the planarian body wall. Almost all planarian muscle cells express PCGs, suggesting a dual function: contraction and control of patterning. PCG expression is dynamic in muscle cells after injury, even in the absence of neoblasts, suggesting that muscle is instructive for regeneration. We conclude that planarian regeneration involves two highly flexible systems: pluripotent neoblasts that can generate any new cell type and muscle cells that provide positional instructions for the regeneration of any body region.

INTRODUCTION

Cellular models for regeneration must explain two essential attributes of adult tissues: the potential for regeneration and the information to guide regeneration. The potential for regeneration refers to the capacity of particular adult cells to replace missing cells (Tanaka and Reddien, 2011). The information for regeneration refers to the molecular instructions that guide which cell types are regenerated. Positional information for regeneration has long been recognized as a key issue (French et al., 1976; Wolpert, 1969), and therefore it is critical to determine the cellular source of adult positional information. Transplantation experiments and Hox gene-expression analyses in vertebrate skin indicate that dermal fibroblasts can influence epithelial positional identity (Dhouailly, 1984; Rinn et al., 2006, 2008). In amphibian limb regeneration, nerves, connective tissue, and epidermis have all been implicated in affecting patterning during regeneration (Nacu et al., 2013; Nacu and Tanaka, 2011). *Hydra* have a body column comprised of ectodermal and endodermal epithelial cells, which possess muscle-like features (myoepithelial cells). *Wnt* genes are expressed in both epithelial layers near the *Hydra* head, and Wnt signaling promotes *Hydra* head regeneration (Broun et al., 2005; Hobmayer et al., 2000; Lengfeld et al., 2009). Despite these advances, how positional identities are established, maintained, and regenerated in adult tissues is poorly understood.

Planarians are flatworms and constitute a classic regeneration model system (Reddien and Sánchez Alvarado, 2004). They can regenerate any missing body part and maintain adult tissues by replacing aging differentiated cells. New cells in planarian regeneration and tissue turnover are produced by neoblasts, adult proliferative cells that include pluripotent stem cells (cNeoblasts) (Reddien and Sánchez Alvarado, 2004; Wagner et al., 2011). The neoblast population therefore harbors the potential for regeneration and tissue turnover. However, it is unknown which cells possess positional information for planarian regeneration. Transplantation of tissues from one body region to another can trigger intercalary regeneration in many regenerative organisms (French et al., 1976; Reddien and Sánchez Alvarado, 2004; Santos, 1931). In intercalary regeneration, missing positional coordinates can be regenerated between juxtaposed tissues, sometimes leading to outgrowths. For example, a cylindrical plug of planarian tissue that has been flipped and inserted (with an inverted dorsoventral [DV] axis) triggers outgrowths (Okada and Sugino, 1937). Irradiation eliminates neoblasts (Dubois, 1949), and yet irradiated DV-inverted plugs still trigger outgrowths in unirradiated hosts, suggesting that positional information might exist in differentiated planarian tissues (Kato et al., 2001).

The molecular basis for positional information (i.e., genes controlling pattern formation) as a biological problem has been investigated primarily in animal embryos and remains understudied in adult tissues. Molecular genetic studies in planarians have revealed that orthologs of numerous embryonic patterning genes in other organisms have roles in adult planarian tissues

CrossMark

for instructing tissue turnover and regeneration (Reddien, 2011). In this study, we define position control genes (PCGs) as genes that (1) display regionalized expression along one or more body axes, and (2) either show a patterning-abnormal RNAi phenotype (e.g., homeotic) or encode a protein that is predicted to regulate pathways (e.g., Wnt, Bmp, or Fgf signaling) that are important for planarian patterning (Reddien, 2011). Most PCGs encode signaling pathway receptors, ligands, or secreted inhibitors. We analyzed more than 20 genes that met these criteria and had expression domains spanning different regions of all body axes. Several examples illustrate PCG properties: *wnt1* is expressed at the animal tail tip and at all wounds, and *wnt1* RNAi causes regeneration of heads in place of tails (Adell et al., 2009; Petersen and Reddien, 2009b), a phenotype that is also observed following RNAi of the Wnt pathway *β-catenin-1* gene (Gurley et al., 2008; Iglesias et al., 2008; Petersen and Reddien, 2008). *notum* is expressed at the anterior pole and anterior-facing wound sites, and *notum* RNAi causes regeneration of tails in place of heads (Petersen and Reddien, 2011). *bmp4* is expressed in a medial-to-lateral messenger RNA (mRNA) gradient on the dorsal side (Orii et al., 1998), and RNAi of *bmp4* causes ventralization (Molina et al., 2007; Orii and Watanabe, 2007; Reddien et al., 2007). In all of these cases, the site of PCG expression corresponds to the body region that is affected by RNAi of that gene. Here, we address the cellular source of the positional information for planarian regeneration by investigating planarian PCGs.

RESULTS

Positional Information Exists in Differentiated Planarian Tissues

A previous study by (Saló and Baguñà, 1985) revealed that tissue regeneration can occur between unirradiated grafts from one anteroposterior (AP) region transplanted into irradiated hosts at a different AP region. In that study, surgical trimming resulted in a wounded, transplanted fragment representing one AP end of the animal (Saló and Baguñà, 1985), and in principle, this wounded fragment end could have influenced the regenerative response. Nonetheless, in this work, we found that cylindrical plugs from the anterior of irradiated animals triggered outgrowth upon transplantation into the posterior of otherwise intact, unirradiated animals (Figures 1A and 1B). The distal outgrowth end expressed an anterior PCG and the proximal end a posterior PCG (Figure 1C). These observations, together with results from additional transplantation experiments (Kato et al., 2001; Saló and Baguñà, 1985), suggest that differentiated planarian cells (nonneoblasts) have positional information that can instruct regenerative responses.

PCG expression patterns define unique domains along the AP, DV, and mediolateral (ML) axes (Reddien, 2011; Figure 1D). Several PCGs have patterning abnormal RNAi phenotypes (Table S1). Other PCGs are homologous to patterning pathway regulators, but do not yet have reported RNAi phenotypes that define their regeneration roles (Table S1). All PCGs examined (Figures 1D, 2A, and S1A) displayed a similar feature: expression in a peripheral, subepidermal cell layer (Figures 2B and S1B; Gaviño and Reddien, 2011; Petersen and Reddien, 2009b, 2011; Reddien et al., 2007) that is devoid of neoblasts (Figures 2B and S1B). Furthermore, irradiation rapidly eliminated neoblasts, but not PCG expression (Figures S2A and S2B; Gaviño and Reddien, 2011; Gurley et al., 2010; Petersen and Reddien, 2009b, 2011; Reddien et al., 2007). These observations indicate that the major site of PCG expression is in non-neoblast cells.

The common subepidermal location of PCG expression raised the possibility that a specific subepidermal cell population plays an informational role in tissue turnover and regeneration. We refer to this candidate cell population as "position control cells" (PCCs), and hypothesize that this PCC population harbors positional information, thus providing body coordinates for maintaining the adult body plan during tissue turnover and guiding regeneration (a biological GPS-like system, with numerous static cell satellites). If this is correct, PCCs should have a broad distribution (to provide information across all body axes, and because regeneration can start from essentially any body fragment), they should be capable of simultaneously expressing multiple patterning factors to define a coordinate system, should dynamically adjust expression during regeneration to produce a new coordinate system, and should not depend on neoblasts for maintenance of PCG expression or for some changes in pattern of expression after injury.

PCGs Are Expressed in the Same Cell Population

To determine whether a PCC population exists, we first asked whether PCGs are expressed together in the same cells. We analyzed three separate PCG pairs by double fluorescence in situ hybridization (FISH) in each of seven zones, which spanned the AP, DV, and ML axes (Figures 2C and S3A). In every case examined, PCGs were coexpressed in the same cells to a substantial degree. For example, 82.7% of *sFRP-2*$^+$ (anterior marker) cells were also *nlg-8*$^+$ (dorsal marker) in the dorsal-head region (zone 1; Figure 2C). Similarly, 73.9% of *sFRP-2*$^+$ zone 1 cells were *ndl-4*$^+$, and 62.6% of *nlg-8*$^+$ zone 1 cells were *ndl-4*$^+$. Similar results were obtained in all seven investigated zones, with coexpression percentages ranging from 24% to 93.6% (Figure 2C). At all analyzed locations, cells coexpressed regulators of multiple signaling pathways and both AP and DV PCGs. These data are consistent with the possible existence of a PCC population.

Planarian Muscle as a Positional Coordinate System

To determine the identity of cells expressing PCGs, we analyzed the relation of cells expressing PCGs *sFRP-2* and *nlg-8* to various known differentiated cell types. *sFRP-2* was not detectably coexpressed in subepidermal neoblast progeny populations (*NB.21.11E*$^+$ and *agat-1*$^+$; Eisenhoffer et al., 2008), peripheral neurons (*chat*$^+$), intestinal cells (*mat*$^+$), or protonephridial tubules (*rootletin*$^+$; Figure 3A). We did, however, identify a cell population with a relatively homogeneous subepidermal distribution resembling putative PCCs, marked by the expression of *collagen* (Figure 3B). We next determined these *collagen*$^+$ cells to be muscle cells, coexpressing muscle markers *troponin* and *tropomyosin* (Figures 3C and 3D). Topologically, planarian muscle nuclei are internal to four layers of muscle fibers, and we found that *collagen*, *tropomyosin*, and *troponin* mRNA

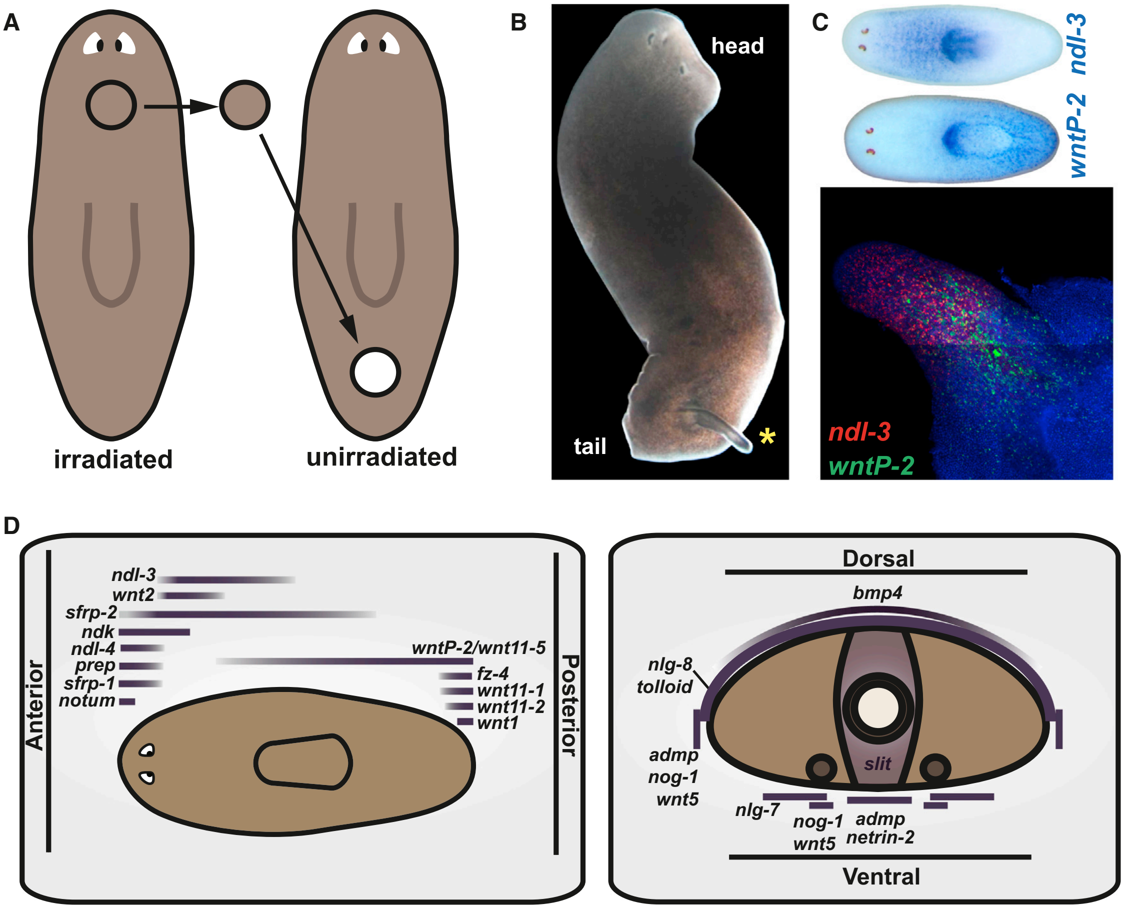

Figure 1. Positional Conflict without Neoblasts

(A) Anterior cylindrical plugs from animals irradiated with 6,000 rad were transplanted into the posterior of unirradiated hosts. For other transplant experiments, see Kato et al., 2001; Saló and Baguñà, 1985.

(B) Tissue outgrowth was triggered by transplantation (yellow asterisk).

(C) *ndl-3* and *wntP-2* were respectively expressed in the anterior and posterior of wild-type animals, and in the distal and proximal regions of an outgrowth.

(D) Cartoon depicting domains of PCG expression, which define positions across AP, DV, and ML body axes (modified from Reddien, 2011).

See also Figure S1.

were concentrated around muscle nuclei, with *troponin* and *tropomyosin* mRNA extending into muscle fibers (Figures 3C and 3D; Movies S1 and S2). The distribution of muscle cells is consistent with the possibility that these cells are PCCs. We therefore tested for coexpression of PCGs and muscle markers.

Every PCG analyzed, including *nlg-8*, *ndl-4*, *sFRP-2*, *ndl-3*, *wntP-2* (also known as *wnt11-5*), *fz-4*, *wnt2*, *nlg-7*, *admp*, *netrin2*, *notum*, *wnt1*, *bmp4*, *wntA*, *wnt5*, *ndk*, *tolloid*, *wnt11-1*, *wnt11-2*, *sFRP-1*, and netrin1, was coexpressed with *troponin* or *collagen* (Figures 3E, S3B, and S3C). The percentage overlap in expression of most PCGs and muscle markers was remarkably high. For example, 99.1% of *nlg-8*$^+$ cells, 96.7% of *wntP-2*$^+$ cells, and 98.8% of *bmp4*$^+$ cells were *troponin*$^+$. PCG mRNA was localized around the nucleus of *troponin*$^+$ cells, possibly because most of these genes encode transmembrane or secreted proteins, which might be localized with perinuclear endoplasmic reticulum-associated ribosomes.

To further demonstrate that cells expressing PCGs are muscle cells, we performed FISH on isolated muscle fibers. We developed an enzymatic tissue-dissociation procedure capable of isolating intact, contractile muscle fibers (Movie S3) with associated offset nuclei (Figure 3F), a known planarian muscle cell morphology (Gelei, 1927). *sFRP-2*, *wntP-2*, and *nlg-8* mRNAs were present around the nuclei of isolated *troponin*$^+$ muscle cells, with some signal also observed in the fiber proximal to the nucleus (Figure 3F). We conclude that PCGs are expressed in muscle cells of unamputated planarians. The PCG expression patterns in muscle described here explain the reported expression patterns for almost every known planarian PCG discovered from extensive RNAi and gene-expression studies over the past 10 years (Adell et al., 2009; Cebrià et al., 2002; Cebrià and Newmark, 2005; Gaviño and Reddien, 2011; Gurley et al., 2008, 2010; Iglesias et al., 2008; Kobayashi et al., 2007; Molina et al., 2007, 2009, 2011; Orii and Watanabe, 2007; Petersen and Reddien, 2008, 2009b,

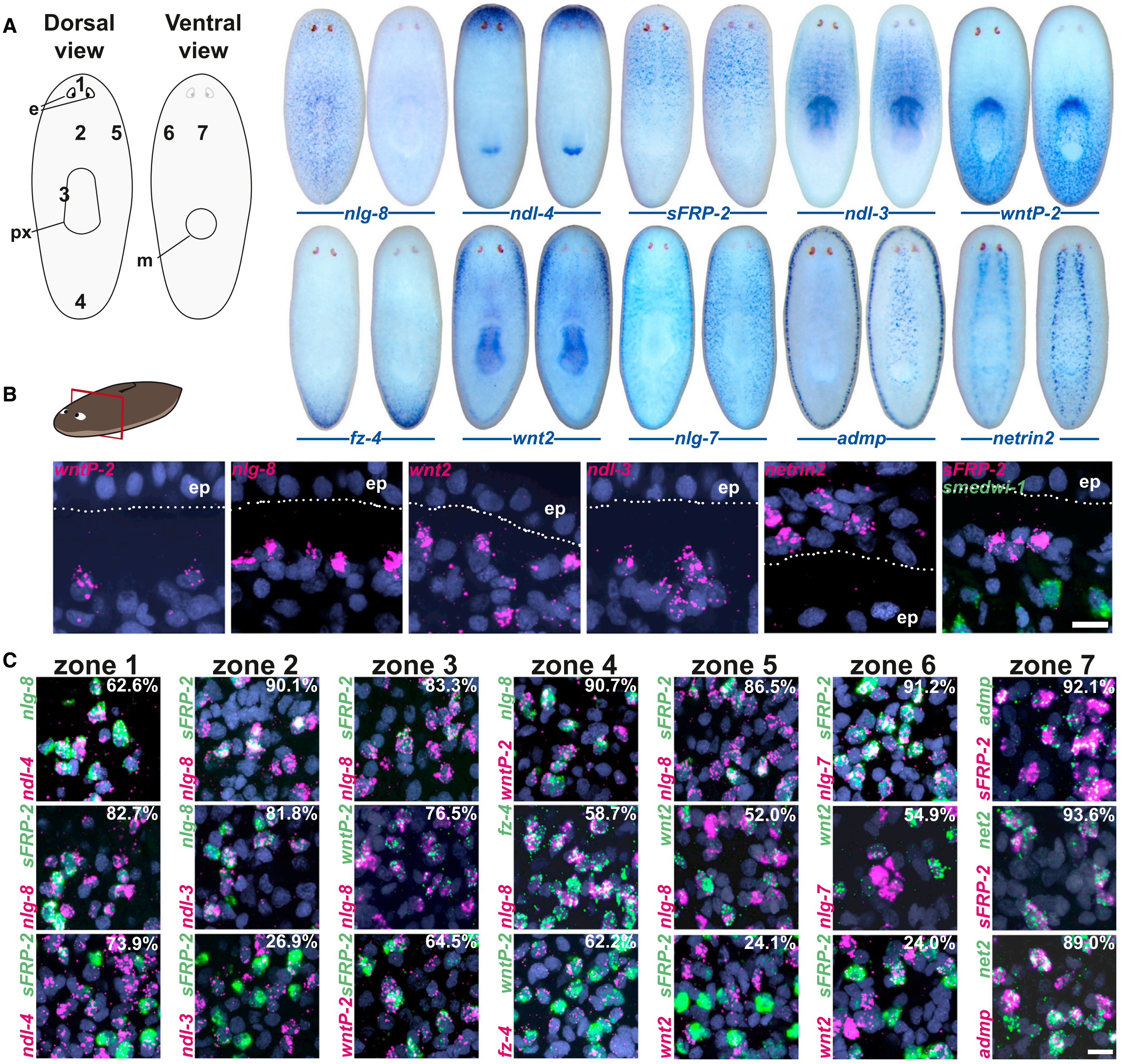

Figure 2. PCGs Are Coexpressed in Subepidermal Cells

(A) Expression patterns of the PCGs analyzed in this figure (see also Figure S1). Cartoon depicts seven analyzed zones (zones 1–7). Anterior: up; left animal: dorsal view; right animal: ventral view. Animals are 1.5–2.5 mm in length. e, eye; m, mouth; px, pharynx.

(B) Fluorescent images of transverse tissue sections. Dorsal up, as illustrated. ep, epidermis.

(C) Three RNA probe pairs were used in double FISH experiments. The percentage of cells expressing the less-abundant (fewer cells) gene that also expressed the more-abundant gene is shown (the less-abundant gene is green in each pair). Three animals were counted and the data were summed for each gene pair. Nuclear signal (DAPI) is shown in blue.

Scale bar, 10 μm (B and C). See also Figures S2 and S3.

2011; Reddien et al., 2005a, 2007; Rink et al., 2009). These findings suggest that muscle is a major site of instructive signaling in planarian regeneration.

To determine whether a minority or majority of muscle cells expressed PCGs, we simultaneously hybridized RNA probes for 19 PCGs and detected the combined signal in the red channel, while detecting *collagen* signal in the green channel. Between 95.7% and 99.8% of muscle cells analyzed in the seven regions described above displayed signal from the combined PCGs (Figures 3G, S3D, and S3E). Therefore, in addition to contraction, expression of PCGs is a major attribute of muscle throughout the planarian body wall.

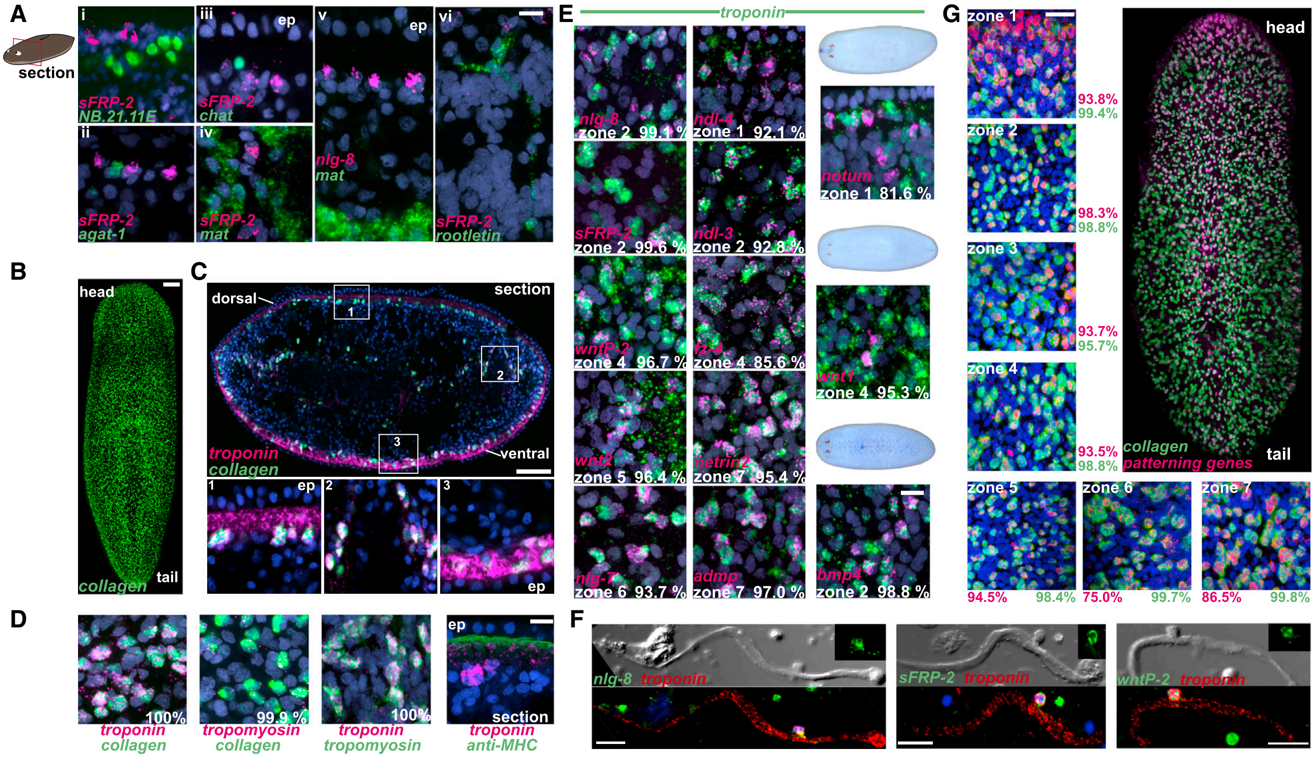

Figure 3. PCGs Are Expressed in Muscle Cells

(A) Prepharyngeal sections (dorsal up; compare with the cartoon) show that position control cells (*sFRP-2*$^+$ or *nlg-8*$^+$) are distinct from specific peripheral neoblast progeny cell types (i, *NB.21.11e*$^+$; ii, *agat-1*$^+$), peripheral neurons (iii, *chat*$^+$), intestinal cells (iv and v, *mat*$^+$), and protonephridia (vi, *rootletin*$^+$). Internal *sFRP-2*$^+$ cells close to intestine (iv) and *nlg-8*$^+$ cells far from intestine (v) are shown. ep, epidermis.

(B) *collagen* is broadly expressed subepidermally, similar to the density and location of position control cells (dorsal view shown; anterior up).

(C) *collagen*$^+$ cells are muscle cells that coexpress *troponin* both subepidermally (insets 1 and 3) and internally (inset 2).

(D) *collagen*$^+$ cells coexpress both *tropomyosin and troponin*. The percentage of *collagen*$^+$ cells (>500 cells counted from three animals each) expressing *troponin*/*tropomyosin* is shown. *troponin* and *tropomyosin* are also coexpressed in the same cells. Right: cross-section showing subepidermal *troponin*$^+$ cells with signal extending into the region of muscle fibers (labeled with the anti-myosin-heavy-chain (MHC) antibody TMUS-13; Cebrià and Vispo, 1997).

(E) PCGs are coexpressed with *troponin* (the percentage of position control cells expressing *troponin* is shown; >250 position control cells were examined from three animals each). Axis regeneration genes *notum* (anterior regeneration), *wnt1* (posterior regeneration), and *bmp4* (dorsal regeneration) were also coexpressed with *troponin* (115, 41, and 327 cells, respectively, were examined). Additional examples are provided in Figure S3B.

(F) Differential interference contrast (DIC, top) and fluorescent micrograph (bottom) of isolated muscle fibers expressing *troponin* and PCGs (six *sFRP-2*$^+$, four *nlg8*$^+$, and four *wntP-2*$^+$ cells were examined). Nuclei (DAPI) are blue. Additional green signal is in debris (lacking a nucleus).

(G) Coexpression of 19 PCGs (red, combined RNA probes) and *collagen* (green) in zones 1–7 (as in Figure 2). The percentage of double-positive cells is given as a fraction of all PCG-positive cells (red) and all *collagen*$^+$ cells (green; three animals examined for each region). Neural *netrin1* and *netrin2* expression affects the percentage of red cells that are *collagen*$^+$ in zones 6 and 7.

Scale bars, 10 μm (A, C [bottom], and D–F), 100 μm (B and C [top]), 200 μm (E), and 20 μm (G). See also Figure S1.

Most PCGs displayed little to no detectable expression outside of subepidermal muscle cells (Figures 3G and S1), but some PCGs were expressed internally. For example, *nlg-8* and *sFRP-2* were expressed in intestinal muscle cells (Figure S3F), several PCGs were expressed in pharyngeal muscle cells (Figure S3G), and *wntP-2* was strongly expressed in muscle cells anterior to the pharynx (Figure S3H). These analyses further demonstrate the capacity of muscle cells to express PCGs, even at locations outside the body wall. Finally, some PCGs also displayed expression in *troponin*$^-$ cells in the brain (*wnt1* and *wntA*, Figure S3I), nerve cords or other neurons (*netrin2*), or in a small ring at the anterior end of the pharynx (*bmp4*; Figure S3J), indicating potential additional functions at other locations for these genes.

Dynamic Change in PCG Expression in Muscle during Regeneration

The concept of GPS-like PCCs could explain the maintenance of regional tissue identities during cell turnover, and the capacity to regenerate missing tissues if PCCs can alter their PCG expression following injury. We therefore examined PCG expression during regeneration. Among numerous known wound-induced genes (Wenemoser et al., 2012) is a class (termed W2) that is induced proximal to wounds and includes several PCGs (e.g., *wnt1*, *wntless*, *glypican-1*, and *notum*) (Adell et al., 2009; Petersen and Reddien, 2009b; Wenemoser et al., 2012). *wnt1*, *wntless*, and *notum* RNAi perturb the head-versus-tail regeneration choice at transverse wounds (Adell et al., 2009; Petersen and Reddien, 2009b, 2011), and

glypican-1 RNAi causes indented head regeneration (Wenemoser et al., 2012), a phenotype similar to that of Bmp-defective planarians. These wound-induced genes (*wnt1*, *wntless*, *glypican-1*, and *notum*), the additional wound-induced genes *inhibin-1* and *nlg-1*, and *bmp4* in parasagittal fragments were all expressed in muscle cells at wounds (Figures 4A, S4A, and S4B). The percentage overlap of expression of W2 genes and muscle markers at wounds was very high. For example, 96.6% of *wnt1*$^+$ cells, 99.7% of *inhibin*$^+$ cells, 100% of *nlg-1*$^+$ cells, and 98% of *notum*$^+$ cells were detectably *collagen*$^+$ at anterior-facing wounds. Tissue sections of wounded animals at multiple body locations showed clear subepidermal, wound-induced expression of *notum* and *wnt1* restricted to *collagen*$^+$ cells (98.4% and 95.7%, respectively, were detectably double positive; Figures 4B, S4C, and S4D). Furthermore, wound-induced expression of the PCGs in muscle cells occurred in irradiated animals, indicating that existing muscle cells at wounds can change their PCG expression profile. Finally, similar to the case for wound-induced PCG expression, cells expressing PCGs in head and tail blastemas were also *troponin*$^+$ (Figure 4C).

We tested whether muscle cells can dynamically adopt new positional identities in response to amputation. Upon amputation, positional information in body fragments must be readjusted to regenerate an appropriately proportioned animal. For example, following transection, tail fragments express anterior-specific PCGs at their anterior end, whereas posterior-specific PCG expression can become posteriorly restricted to the tail fragment tip (Gurley et al., 2010; Petersen and Reddien, 2009a). Some of these expression changes specific to anterior or posterior regeneration also occur in the absence of neoblasts (Gurley et al., 2010; Petersen and Reddien, 2009a). We examined expression of *wnt2*, which is expressed in the head, and *wntP-2*, which is expressed in a broad posterior-to-anterior gradient emanating from the tail tip (Figures 1D and S1), utilizing amputated tail fragments that had been previously irradiated to prevent new cell formation. Following amputation, *wnt2* was expressed in anterior muscle cells of the tail fragment and *wntP-2* expression receded to muscle cells of the tail tip (Figure 4D). The expression domains of these genes were thus partially regenerated in the existing muscle cells of the irradiated tail fragments, which were incapable of new tissue regeneration. A considerable fraction of anterior *collagen*$^+$ muscle cells showed both new *wnt2* expression and retained *wntP-2* expression (45.6% at 2 days and 66.4% at 4 days; Figure 4D). Coexpression of *wnt2* and *wntP-2* further indicated that previously posterior muscle cells can express anterior PCGs while simultaneously decreasing posterior PCGs after amputation.

Finally, we assessed neoblast lineage specification following RNAi of genes that are expressed in muscle at wound sites: *wnt1* and *notum. smedwi-1* is expressed specifically in neoblasts (Reddien et al., 2005b) and *ovo* is expressed in a subset of *smedwi-1*$^+$ neoblasts at anterior-facing wounds that are progenitors for eye regeneration (Lapan and Reddien, 2012). *ovo*$^+$/*smedwi*$^+$ cells were present at posterior-facing wounds following *wnt1* RNAi, but not in the control (4 days postamputation [4 dpa]; Figures 4E and S4E). Conversely, smaller than normal numbers of *ovo*$^+$ cells, including *ovo*$^+$/*smedwi*$^+$ cells, were present at anterior-facing wounds in *notum(RNAi)* animals (Figure S4F). These data demonstrate that the fates of at least some neoblasts are dependent on *wnt1* and *notum*, which were induced in muscle at wounds (Figures 4A, 4B, S4A, and S4C). Whether this reflects direct action on neoblasts by WNT1/NOTUM proteins is unknown. Because PCGs can be instructive for regeneration (e.g., *wnt1* and *notum*) and some PCG expression domains can be regenerated in existing muscle tissue in irradiated amputated animals, we propose that changes in muscular PCG expression functions instructively for downstream regeneration programs (Figure 4F).

DISCUSSION

Positional information is essential for maintaining regional tissue identity during tissue turnover and wound repair in most adult animals, but it remains poorly understood. Planarians represent an ideal system for molecular and cellular investigations of adult positional information because of their extensive tissue turnover and regeneration, and because planarian gene function can readily be studied in adults via RNAi and in situ hybridizations. In principle, adult tissue identity could be retained during tissue turnover by cell populations that autonomously maintain their regional identity (e.g., stem cells). By contrast, genes that are expressed regionally in differentiated adult cells could regulate one another through signaling pathways such that a stable pattern is maintained and influences the fate of resident stem cells. Our data from planarians favor the latter model: patterns of PCG expression exist in adult muscle cells, but not in neoblasts, and these patterns are maintained in adult life and facilitate scalability during growth. Besides muscle, other cell types likely have roles in planarian regeneration. Which cells and signals regulate neoblast maintenance, proliferation, migration to wounds, and specialization remains largely unknown. Regardless, our results demonstrate that almost all of the planarian PCG expression patterns previously reported—for genes with roles ranging from maintaining the DV axis (*bmp*) to mediating the head-tail regeneration choice (*wnt1* and *notum*)—are localized in muscle, indicating that subepidermal planarian muscle tissue is a major source of the positional information that guides tissue turnover and regeneration programs.

Injuries remove local patterning instructions, requiring the pattern of PCG expression in muscle itself to be regenerated. We propose a model in which expression changes in existing muscle cells occur as an early and instructive process in regeneration, with positional information being dynamic in an initially static set of muscle cells (Figure 4F). In this model, new PCG expression in muscle at wounds influences (directly or indirectly) the cell types made by neoblasts, promoting regeneration of missing cell types (Figure 4F). How the PCG pattern in muscle is reestablished after injury is largely unknown, but events involved in the head-versus-tail regeneration choice (regeneration polarity) at transverse wounds are the best understood (Gurley et al., 2008, 2010; Iglesias et al., 2008; Petersen and Reddien, 2008, 2009b, 2011). The previously described mechanism for regeneration polarity can be viewed together with the findings reported here in a model for AP axis regeneration: wound signaling generically activates *wnt1* expression in muscle cells at wounds, giving any wound the chance to regenerate a tail

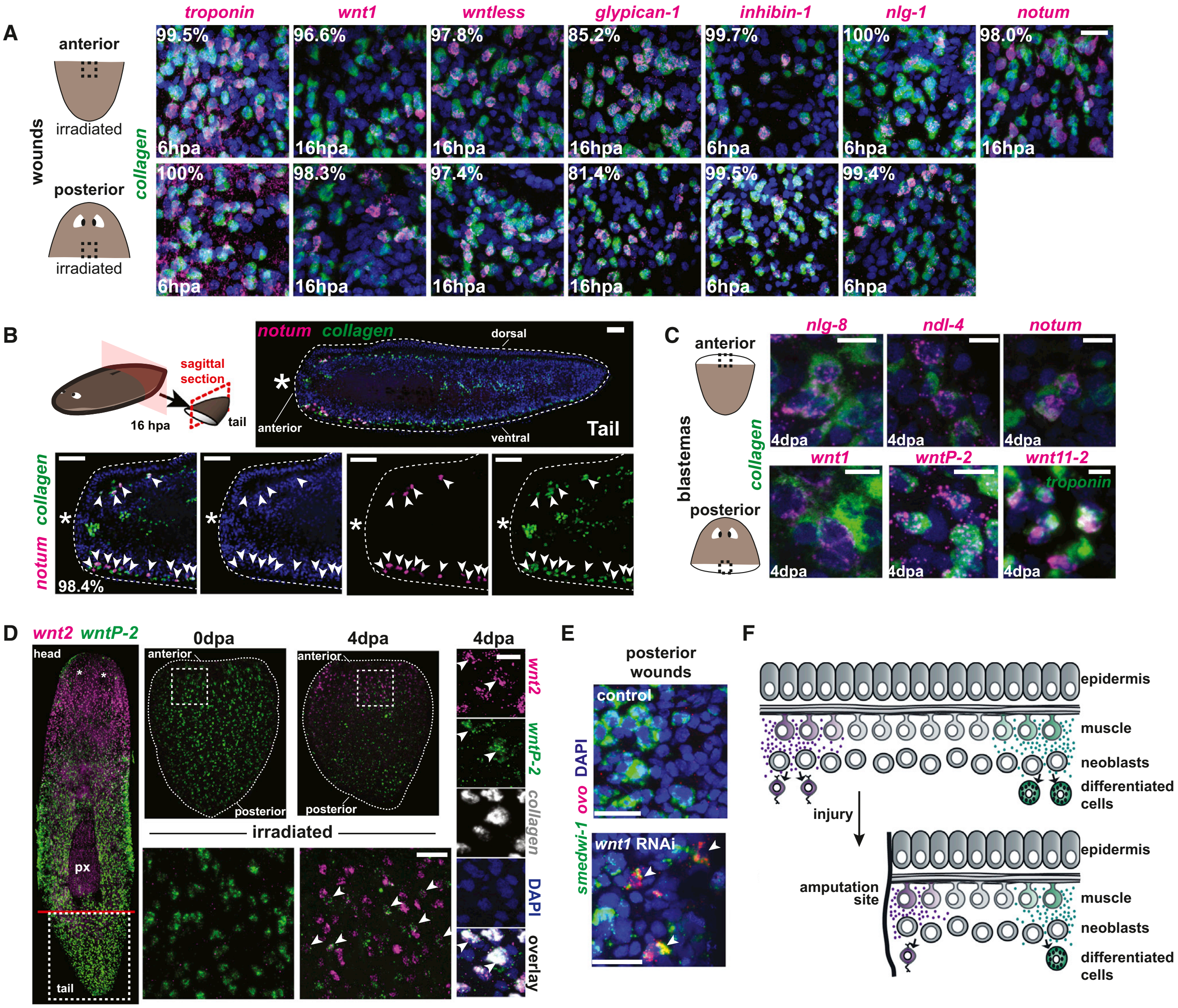

Figure 4. Dynamic PCG Expression in Muscle Cells following Amputation

(A) Animals were irradiated with 6,000 rads (eliminating neoblasts) and amputated transversely 3 days later. The percentage of PCG^{+} cells at wounds expressing *collagen* or *troponin* was examined in three animals each. Anterior-facing wounds are from tails, and posterior-facing wounds are from heads (except for *nlg-1* and *glypican*, where one anterior-facing wound was from a trunk; 117–547 cells were examined for each condition); 108/108 and 423/425 *collagen*$^{+}$ cells were *troponin*$^{+}$ at anterior- and posterior-facing wounds, respectively, at 6 hours postamputation (6 hpa; 168/168 and 141/141 were double positive at 16 hpa).

(B) Schematic illustrating the surgical procedure. Tail fragments were fixed at 16 hpa, labeled by FISH and DAPI, and analyzed in sagittal sections. Anterior is left. Bottom: zoomed images from the anterior-facing wound site. Arrowheads indicate coexpression of *notum* and *collagen* in cells adjacent to the amputation plane (n = 418/425 were double positive).

(C) PCGs with regeneration RNAi phenotypes are coexpressed with *collagen* or *troponin* in blastemas (the time after amputation is indicated).

(D) Transient coexpression of anterior-specific (*wnt2*) and posterior-specific (*wntP-2*) PCGs during regeneration. Animals were irradiated (6,000 rads) and amputated 3 days later. Left: intact animal (3 days postirradiation). Center: tail fragments (bottom, zoomed) at 0 dpa and 4 dpa. *wnt2* was coexpressed with *wntP-2* at 4 dpa. Right: Triple-color FISH demonstrates coexpression of *wnt2*, *wntP-2*, and *collagen*.

(E) *wnt1(RNAi)* animals had clusters of numerous *ovo*$^{+}$ cells at posterior-facing wounds at 4 dpa (n = 2/5 animals, with three total clusters present and none present in control RNAi [n = 4] animals; see also Figure S4E). All posterior *ovo*$^{+}$ cell clusters in *wnt1(RNAi)* animals possessed multiple *smedwi-1*$^{+}$/*ovo*$^{+}$ cells (arrowheads; *smedwi-1* expression marks neoblasts).

(F) Model. PCG expression in muscle specifies the identity of new cell types made in tissue turnover. Following amputation, muscle cells change their PCG expression, and these changes dictate which type of new tissue is regenerated. The influence of muscle on neoblasts is depicted by purple and green signaling environments, but it need not be direct.

Scale bars, 20 μm (A), 50 μm (B), and 10 μm (C and D). See also Figure S1.

if no posterior tissue juxtaposes the wound. At anterior-facing wounds, *notum* is activated in muscle cells and inhibits Wnt signaling to give any anterior-facing wound the opportunity to regenerate a head if it is not juxtaposed by anterior tissue. Initiation of head or tail regeneration is followed by pattern restoration involving the rescaling of existing PCG gradients in muscle (e.g., *wntP-2/wnt11-5* in tail fragments) and the emergence of missing patterns of gene expression, likewise in muscle (e.g., *wnt2* in tail fragments). DV and ML patterns must also be regenerated, and the genes involved in determining these axes also show dynamic expression in muscle. We propose that a combination of two flexible cell types—muscle cells that are capable of expressing position control genes for any body region, and cNeoblasts that are capable of generating all differentiated cell types—enables the dramatic ability of planarians to regenerate any missing body part.

EXPERIMENTAL PROCEDURES

Fixations, In Situ Hybridizations, and Immunostainings

Nitroblue tetrazolium/5-bromo-4-chloro-3-indolyl phosphate (NBT/BCIP) colorimetric in situ hybridization and FISH were performed as previously described (Pearson et al., 2009). Animals were placed in 5% N-acetyl-cysteine in 1× PBS for 5 min at room temperature followed by fixation in 4% formaldehyde. Animals were bleached in 6% hydrogen peroxide overnight and stored in methanol at −20°C. Digoxigenin (DIG)-, fluorescein-, and dinitrophenol (DNP)-labeled riboprobes were synthesized as previously described (Pearson et al., 2009). For double and triple labeling, horseradish peroxidase (HRP) inactivation was performed for 45 min between labelings in 4% formaldehyde diluted in PBSTx (0.1% Triton X-100). Some HRP inactivations were performed overnight (≥16 hr) in 1% w/v (154 mM) Na Azide dissolved in PBSTx. To detect the expression of multiple PCGs at the same time in the same channel (Figure 3G), DIG probes against 19 GPS genes (*ndk*, *ndl-4*, *ndl-3*, *notum*, *sFRP-1*, *sFRP-2*, *wnt2*, *wntA*, *wnt11-1*, *wnt11-2*, *wntP-2*, *wnt1*, *wnt5*, *bmp4*, *nlg-7*, *slit*, *admp*, *netrin1*, and *netrin2*) were hybridized simultaneously, together with a DNP probe against *collagen*. After mRNA labeling was completed, samples were blocked for immunolabeling when used in PBSTB (0.3% Triton X-100, 0.25% BSA) and incubated overnight at room temperature in the monoclonal antibody TMUS13 (kind gift of Dr. Rafael Romero) diluted 1:10 in PBSTB. Linked primary TMUS13 antibody was detected with a secondary Alexa488-coupled anti-mouse antibody (1:650 in PBSTB). Gene model sequences for *collagen* (SMED_00066_V2), *troponin* (SMED_00109_V2), and *tropomyosin* (SMED_00440_V2) are available at GEO (GPL14150, see Supplementary File GPL14150_gene_models.txt).

Microscopy and Image Analysis

Fluorescent micrographs were acquired using the Zen software on a Zeiss LSM 700 laser scanning confocal microscope and 405 nm, 488 nm, 555 nm, and 639 nm laser lines. Image analysis was performed using ImageJ and Fiji. To determine the percentage of coexpression of multiple PCGs, cells were quantified in triplicate from stacks within defined regions of each of seven zones. Cells were counted using the Cell Counter plugin (http://rsbweb.nih.gov/ij/plugins/cell-counter.html).

Enzymatic Preparation of Individual Muscle Fibers

Whole worms were amputated into sagittal strips in CMF (15 mM HEPES, 400 mg/l NaH_2PO_4, 800 mg/l NaCl, 1,200 mg/l KCl, 800 mg/l $NaHCO_3$, 240 mg/l glucose, 1% BSA, pH 7.3). Fragments were placed in 100 μl of CMF together with a 1:10 dilution of Liberase TH (Roche), an enzymatic mix of collagenase I and II, and a neutral protease, and incubated at 30°C. Mechanical tissue loosening involved gentle tube flicking and slow pipetting with a cut pipet tip approximately every 15–20 min. Too vigorous mechanical manipulation resulted in shearing of fibers; mixing frequency was determined based upon tissue appearance using Nomarski optics during dissociation. Tissues were incubated in liberase in CMF for 45 min to 1.5 hr, depending on the starting fragment size and handling, until suitable dissociation occurred.

RNAi

Animals were injected with *notum* and *wnt1* double-stranded RNA (dsRNA) after head and tail amputation, injected again the next day, and then left to rest for 1 day. This 3-day procedure was repeated two consecutive times, and the animals were fixed 48 hr or 4 days after the final amputation. *Caenorhabditis elegans unc-22* dsRNA served as the RNAi control.

SUPPLEMENTAL INFORMATION

Supplemental Information includes four figures, one table, and three movies and can be found with this article online at http://dx.doi.org/10.1016/j.celrep.2013.07.022.

ACKNOWLEDGMENTS

We thank Neena Pyzocha for *collagen*-labeled section observations, Irving Wang for illustration assistance, and the entire Reddien laboratory for support and discussions. This work was supported by NIH R01GM080639, the Keck Foundation, and a Helen Hay Whitney postdoctoral fellowship to M.M.

Received: March 26, 2013
Revised: June 24, 2013
Accepted: July 17, 2013
Published: August 15, 2013

REFERENCES

Adell, T., Salò, E., Boutros, M., and Bartscherer, K. (2009). *Smed-Evi/Wntless* is required for beta-catenin-dependent and -independent processes during planarian regeneration. Development *136*, 905–910.

Broun, M., Gee, L., Reinhardt, B., and Bode, H.R. (2005). Formation of the head organizer in hydra involves the canonical Wnt pathway. Development *132*, 2907–2916.

Cebrià, F., and Vispo, M. (1997). Myocyte differentiation and body wall muscle regeneration in the planarian *Girardia tigrina*. Dev. Genes Evol. *207*, 306–316.

Cebrià, F., and Newmark, P.A. (2005). Planarian homologs of netrin and netrin receptor are required for proper regeneration of the central nervous system and the maintenance of nervous system architecture. Development *132*, 3691–3703.

Cebrià, F., Kobayashi, C., Umesono, Y., Nakazawa, M., Mineta, K., Ikeo, K., Gojobori, T., Itoh, M., Taira, M., Sánchez Alvarado, A., et al. (2002). *nou-darake*, a novel gene related to FGF receptors is involved in restricting brain tissues to the head region of planarians. Nature *419*, 620–624.

Dhouailly, D. (1984). Pattern Formation, a Primer in Developmental Biology (New York: Macmillan).

Dubois, F. (1949). Contribution à l'étude de la migration des cellules de régénération chez les *Planaires dulcicoles*. Bull. Biol. Fr. Belg. *83*, 213–283.

Eisenhoffer, G.T., Kang, H., and Sánchez Alvarado, A. (2008). Molecular analysis of stem cells and their descendants during cell turnover and regeneration in the planarian *Schmidtea mediterranea*. Cell Stem Cell *3*, 327–339.

French, V., Bryant, P.J., and Bryant, S.V. (1976). Pattern regulation in epimorphic fields. Science *193*, 969–981.

Gaviño, M.A., and Reddien, P.W. (2011). A Bmp/Admp regulatory circuit controls maintenance and regeneration of dorsal-ventral polarity in planarians. Curr. Biol. *21*, 294–299.

Gelei, J. (1927). Der Bau der Tricladen Muskulatur. Zool. Anz. *73*, 21–32.

Gurley, K.A., Rink, J.C., and Sánchez Alvarado, A. (2008). Beta-catenin defines head versus tail identity during planarian regeneration and homeostasis. Science *319*, 323–327.

Gurley, K.A., Elliott, S.A., Simakov, O., Schmidt, H.A., Holstein, T.W., and Sánchez Alvarado, A. (2010). Expression of secreted Wnt pathway

components reveals unexpected complexity of the planarian amputation response. Dev. Biol. *347*, 24–39.

Hobmayer, B., Rentzsch, F., Kuhn, K., Happel, C.M., von Laue, C.C., Snyder, P., Rothbächer, U., and Holstein, T.W. (2000). WNT signalling molecules act in axis formation in the diploblastic metazoan Hydra. Nature *407*, 186–189.

Iglesias, M., Gomez-Skarmeta, J.L., Saló, E., and Adell, T. (2008). Silencing of *Smed-betacatenin1* generates radial-like hypercephalized planarians. Development *135*, 1215–1221.

Kato, K., Orii, H., Watanabe, K., and Agata, K. (2001). Dorsal and ventral positional cues required for the onset of planarian regeneration may reside in differentiated cells. Dev. Biol. *233*, 109–121.

Kobayashi, C., Saito, Y., Ogawa, K., and Agata, K. (2007). Wnt signaling is required for antero-posterior patterning of the planarian brain. Dev. Biol. *306*, 714–724.

Lapan, S.W., and Reddien, P.W. (2012). Transcriptome analysis of the planarian eye identifies *ovo* as a specific regulator of eye regeneration. Cell Rep. *2*, 294–307.

Lengfeld, T., Watanabe, H., Simakov, O., Lindgens, D., Gee, L., Law, L., Schmidt, H.A., Ozbek, S., Bode, H., and Holstein, T.W. (2009). Multiple Wnts are involved in Hydra organizer formation and regeneration. Dev. Biol. *330*, 186–199.

Molina, M.D., Saló, E., and Cebrià, F. (2007). The BMP pathway is essential for re-specification and maintenance of the dorsoventral axis in regenerating and intact planarians. Dev. Biol. *311*, 79–94.

Molina, M.D., Saló, E., and Cebrià, F. (2009). Expression pattern of the expanded noggin gene family in the planarian *Schmidtea mediterranea*. Gene Expr. Patterns *9*, 246–253.

Molina, M.D., Neto, A., Maeso, I., Gómez-Skarmeta, J.L., Saló, E., and Cebrià, F. (2011). *Noggin* and *noggin-like* genes control dorsoventral axis regeneration in planarians. Curr. Biol. *21*, 300–305.

Nacu, E., and Tanaka, E.M. (2011). Limb regeneration: a new development? Annu. Rev. Cell Dev. Biol. *27*, 409–440.

Nacu, E., Glausch, M., Le, H.Q., Damanik, F.F., Schuez, M., Knapp, D., Khattak, S., Richter, T., and Tanaka, E.M. (2013). Connective tissue cells, but not muscle cells, are involved in establishing the proximo-distal outcome of limb regeneration in the axolotl. Development *140*, 513–518.

Okada, Y.K., and Sugino, H. (1937). Transplantation experiments in planaria *gonocephala Duges*. Zoological Institute. Kyoto Imperial University *7*, 373–439.

Orii, H., and Watanabe, K. (2007). Bone morphogenetic protein is required for dorso-ventral patterning in the planarian *Dugesia japonica*. Dev. Growth Differ. *49*, 345–349.

Orii, H., Kato, K., Agata, K., and Watanabe, K. (1998). Molecular cloning of bone morphogenetic protein (BMP) gene from the planarian *Dugesia japonica*. Zool. Sci. *15*, 871–877.

Pearson, B.J., Eisenhoffer, G.T., Gurley, K.A., Rink, J.C., Miller, D.E., and Sánchez Alvarado, A. (2009). Formaldehyde-based whole-mount in situ hybridization method for planarians. Dev. Dyn. *238*, 443–450.

Petersen, C.P., and Reddien, P.W. (2008). *Smed-betacatenin-1* is required for anteroposterior blastema polarity in planarian regeneration. Science *319*, 327–330.

Petersen, C.P., and Reddien, P.W. (2009a). Wnt signaling and the polarity of the primary body axis. Cell *139*, 1056–1068.

Petersen, C.P., and Reddien, P.W. (2009b). A wound-induced Wnt expression program controls planarian regeneration polarity. Proc. Natl. Acad. Sci. USA *106*, 17061–17066.

Petersen, C.P., and Reddien, P.W. (2011). Polarized notum activation at wounds inhibits Wnt function to promote planarian head regeneration. Science *332*, 852–855.

Reddien, P.W. (2011). Constitutive gene expression and the specification of tissue identity in adult planarian biology. Trends Genet. *27*, 277–285.

Reddien, P.W., and Sánchez Alvarado, A. (2004). Fundamentals of planarian regeneration. Annu. Rev. Cell Dev. Biol. *20*, 725–757.

Reddien, P.W., Bermange, A.L., Murfitt, K.J., Jennings, J.R., and Sánchez Alvarado, A. (2005a). Identification of genes needed for regeneration, stem cell function, and tissue homeostasis by systematic gene perturbation in planaria. Dev. Cell *8*, 635–649.

Reddien, P.W., Oviedo, N.J., Jennings, J.R., Jenkin, J.C., and Sánchez Alvarado, A. (2005b). SMEDWI-2 is a PIWI-like protein that regulates planarian stem cells. Science *310*, 1327–1330.

Reddien, P.W., Bermange, A.L., Kicza, A.M., and Sánchez Alvarado, A. (2007). BMP signaling regulates the dorsal planarian midline and is needed for asymmetric regeneration. Development *134*, 4043–4051.

Rink, J.C., Gurley, K.A., Elliott, S.A., and Sánchez Alvarado, A. (2009). Planarian Hh signaling regulates regeneration polarity and links Hh pathway evolution to cilia. Science *326*, 1406–1410.

Rinn, J.L., Bondre, C., Gladstone, H.B., Brown, P.O., and Chang, H.Y. (2006). Anatomic demarcation by positional variation in fibroblast gene expression programs. PLoS Genet. *2*, e119.

Rinn, J.L., Wang, J.K., Allen, N., Brugmann, S.A., Mikels, A.J., Liu, H., Ridky, T.W., Stadler, H.S., Nusse, R., Helms, J.A., and Chang, H.Y. (2008). A dermal HOX transcriptional program regulates site-specific epidermal fate. Genes Dev. *22*, 303–307.

Saló, E., and Baguñà, J. (1985). Proximal and distal transformation during intercalary regeneration in the planarian *Dugesia (S) mediterranea*. Rouxs Arch. Dev. Biol. *194*, 364–368.

Santos, F.V. (1931). Studies on transplantation in planaria. Physiol. Zool. *4*, 111–164.

Tanaka, E.M., and Reddien, P.W. (2011). The cellular basis for animal regeneration. Dev. Cell *21*, 172–185.

Wagner, D.E., Wang, I.E., and Reddien, P.W. (2011). Clonogenic neoblasts are pluripotent adult stem cells that underlie planarian regeneration. Science *332*, 811–816.

Wenemoser, D., Lapan, S.W., Wilkinson, A.W., Bell, G.W., and Reddien, P.W. (2012). A molecular wound response program associated with regeneration initiation in planarians. Genes Dev. *26*, 988–1002.

Wolpert, L. (1969). Positional information and the spatial pattern of cellular differentiation. J. Theor. Biol. *25*, 1–47.

Supplemental Information

SUPPLEMENTAL REFERENCES

Cebrià, F., Guo, T., Jopek, J., and Newmark, P.A. (2007). Regeneration and maintenance of the planarian midline is regulated by a slit orthologue. Dev. Biol. *307*, 394–406.

Glazer, A.M., Wilkinson, A.W., Backer, C.B., Lapan, S.W., Gutzman, J.H., Cheeseman, I.M., and Reddien, P.W. (2010). The Zn finger protein Iguana impacts Hedgehog signaling by promoting ciliogenesis. Dev. Biol. *337*, 148–156.

Marsal, M., Pineda, D., and Saló, E. (2003). *Gtwnt-5* a member of the wnt family expressed in a subpopulation of the nervous system of the planarian *Girardia tigrina*. Gene Expr. Patterns *3*, 489–495.

Wagner, D.E., Ho, J.J., and Reddien, P.W. (2012). Genetic regulators of a pluripotent adult stem cell system in planarians identified by RNAi and clonal analysis. Cell Stem Cell *10*, 299–311.

Wenemoser, D., and Reddien, P.W. (2010). Planarian regeneration involves distinct stem cell responses to wounds and tissue absence. Dev. Biol. *344*, 979–991.

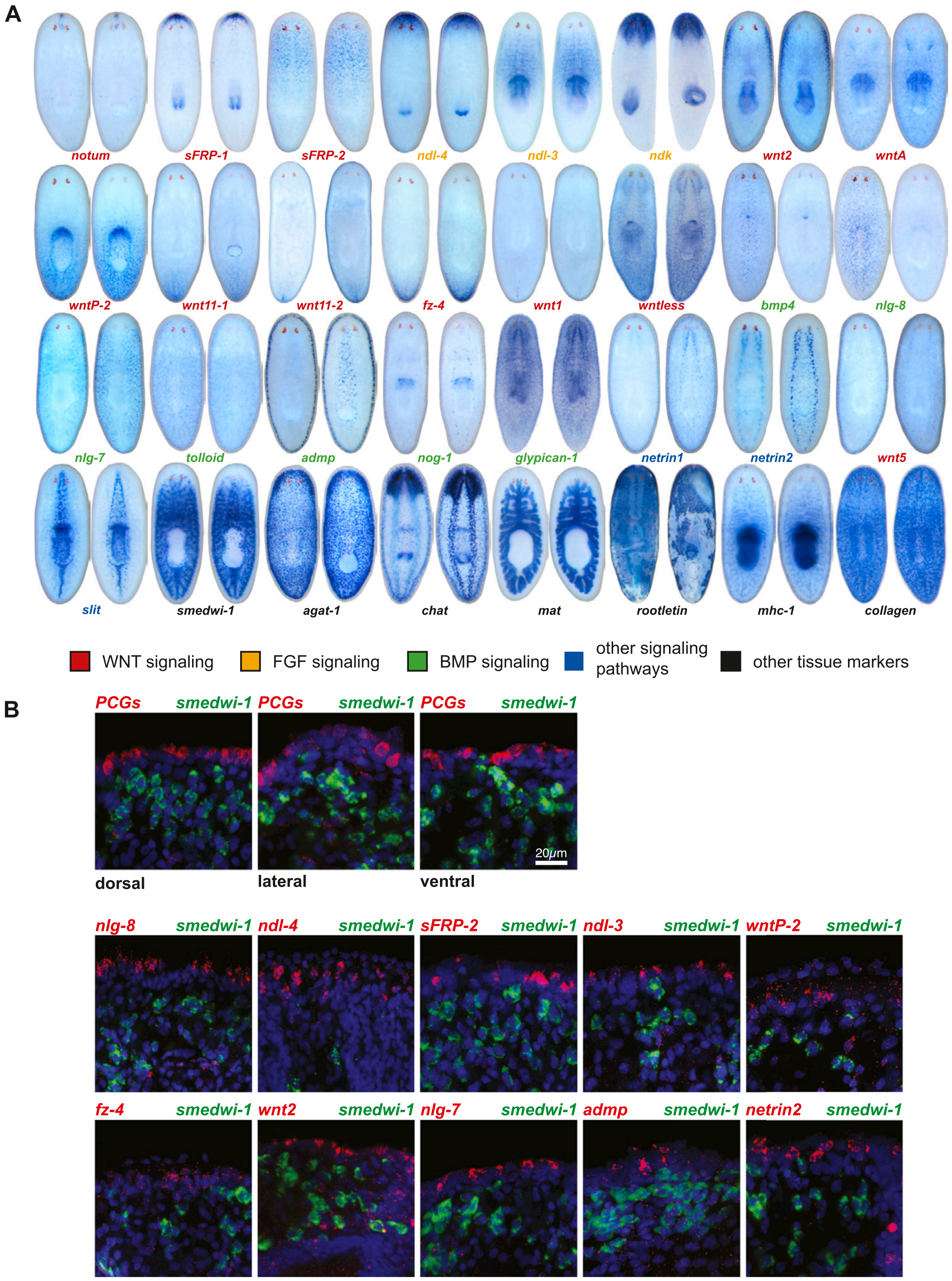

Figure S1. Expression Patterns of the PCG Genes Used in this Study, Related to Figures 1, 2, 3, and 4

(A) In situ hybridizations on wild-type animals with PCG genes and other anatomical markers used in this manuscript. For each gene, two images are shown. Left, dorsal view; right, ventral view. Anterior is to the top in all images; all animals are 1.5-2.5mm in length. The color of the labeling indicates which signaling pathway the gene is part of. In addition to PCGs, the figure also depicts the expression pattern of other tissue markers used in this manuscript, namely *smedwi-1* (Reddien et al., 2005b), *agat-1* (Eisenhoffer et al., 2008), *chat* (Wagner et al., 2011), *mat* (Wenemoser and Reddien, 2010), and *mhc-1*. *rootletin* (Glazer et al., 2010) was used to mark nephridia in Figure 3.

(B) Position control genes are expressed in a subepidermal layer, peripheral to neoblasts. Top row: prepharyngeal sections showing the subepidermal localization of GPS cells marked by the combined expression of 10 position control genes (PCGs; the same as used in Figure 2A) in red and the more interiorly localized neoblasts marked by the expression of *smedwi-1* in green. Bottom two rows: Sections showing the expression of *smedwi-1* in green and individual position control genes in red in the respective regions of their expression. Note that in most cases shown here, the epidermis is missing (due to the experimental procedure).

Cell PRESS Open ACCESS

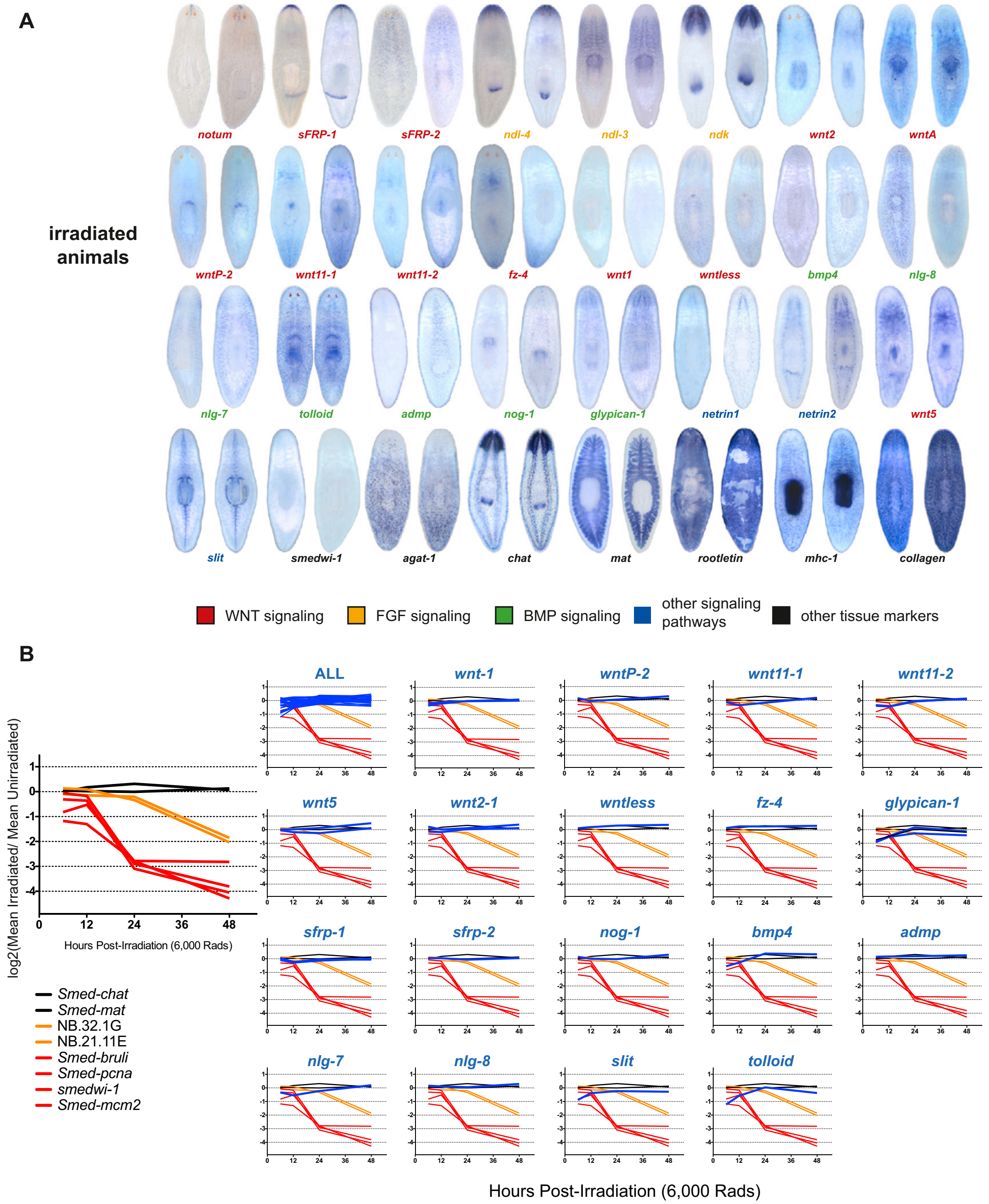

Figure S2. PCGs Are Not Expressed in Neoblasts, Related to Figure 2

(A) Position control gene expression is not irradiation sensitive. For each gene, two images are shown. Left, dorsal view; right, ventral view. Anterior is to the top in all images; all animals are 1.5-2.5mm in length. Animals were irradiated with 6,000 rad and fixed three days later. Animals were then labeled with all markers used in this manuscript. *smedwi-1* is expressed in neoblasts, and lack of *smedwi-1* + cells demonstrates irradiated eliminated neoblasts.

(B) Effects of irradiation on PCG expression levels. Levels of PCG gene expression in whole animal tissues 12, 24, 36, and 48 hr post-irradiation (6,000 Rads) were obtained from microarray data sets available in GEO, accession number GSE34969 (Wagner et al., 2012). Expression levels are represented as log2 values of mean intensity ratios (irradiated/unirradiated). Left, Shown are control gene expression levels for differentiated tissues (*Smed-chat* and *Smed-mat*), which remain unchanged following irradiation. Also shown are representative markers of neoblasts (*Smed-bruli, Smed-pcna, smedwi-1, Smed-mcm2*) and neoblast progeny cell types (NB.32.1G, NB,21.11E), which are rapidly depleted within 48 hr of irradiation. Right, Expression profiles of PCGs following irradiation. Control genes use same color scheme as at left.

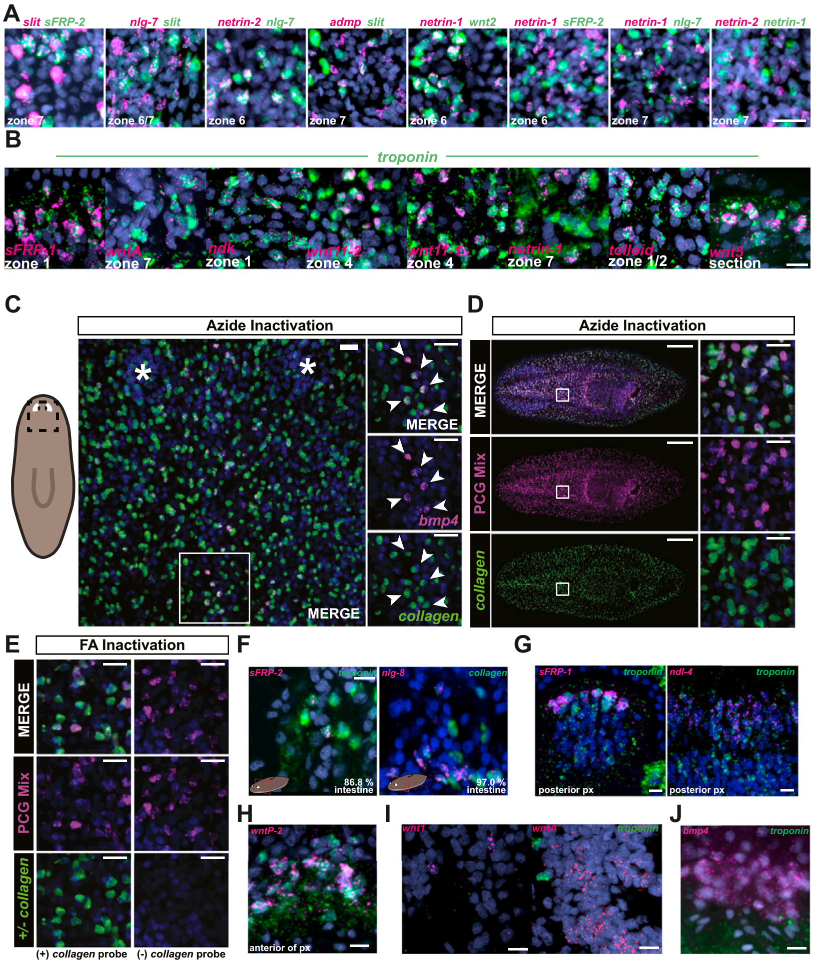

Figure S3. Analysis of PCG Expression, with Controls for FISH, Related to Figures 2 and 3

(A) *wnt2/netrin1*, *netrin2/nlg-7*, *nlg-7/slit, netrin2/nlg-7*, and *admp/slit* displayed overlapping expression in the same cells. Overlap at lower levels was observed for *netrin1/sFRP-2*, *netrin1/nlg-7*, and little overlap was observed between *netrin1/netrin2*. Therefore, a subpopulation of position control cells expresses *netrin2/sFRP-2/nlg-7* at high frequencies, and another coexpresses *netrin1/wnt2* at high frequencies, with some overlap between these two populations. Scale bar, 10 μm.

(B) Additional examples of position control genes expressed in muscle. Position control genes are coexpressed with *troponin*. The zone in which the image was taken is indicated (compare schematic in Figure 2). For *wnt5* expression, lateral expression in a transverse tissue section is shown. Scale bar, 10 μm.

(C–E) Controls for fluorescent in situ hybridizations. Shown are representative confocal projections from whole-mount fluorescent in situ hybridization (FISH) experiments. DIG probes were developed first using anti-DIG-POD and rhodamine tyramide (magenta). Peroxidase activity was then quenched using either 1% sodium azide or 4% formaldehyde in PBSTx (see methods). Fluorescein probes were developed second using anti-Fluor-POD and FITC tyramide (green), and DAPI stained (blue).

(C) Representative dorsal projection (anterior up) showing *bmp4* expressing cells expressing *collagen*. Scale bars, 20 μm. Asterisks indicate pigment cups. Representative ventral projection, anterior left.

(D) Coexpression of position control genes (PCGs) *ndk, ndl-3, ndl-4, notum, sFRP2, wnt2, wntA, wnt11-1, wnt11-2, wntP-2, wntP-1, wnt5, bmp4, nlg7, slit, admp* (magenta) with *collagen* (green) is shown. Scale bars 200 μm (left), 20 μm (insets).

(E) Representative confocal projections from animals labeled for PCG expression as in (D) using the 4% formaldehyde inactivation method. The extent of green fluorescence due to residual HRP activity following inactivation is shown in the right column of images (*collagen* probe omitted). Left and right image panels were collected with identical pinhole, laser, and gain settings on a Zeiss LSM 700 scanning confocal microscope.

(F–J) Positional control gene expression outside the subepidermal layer. Scale bar, 10 μm.

(F) Tissue sections (see schematic inset) display *sFRP-2* and *nlg-8* expression around the intestine, and these cells also express *troponin/collagen*. Percentage of position control gene$^+$ cells expressing *troponin/collagen* is shown (>500 total position control gene$^+$ cells examined from three animals each).

(G) *sFRP-1* and *ndl-4* are expressed within the pharynx in pharyngeal muscle cells (*troponin*$^+$).

(H) *wntP-2* is expressed strongly internally at the anterior end of the pharynx in cells that also express *troponin*.

(I) *wnt1* and *wntA* display brain region expression that is not in muscle cells.

(J) *bmp4* is expressed in a small ring of *troponin*$^-$ cells at the anterior end of the pharynx.

(G–J) Anterior, up.

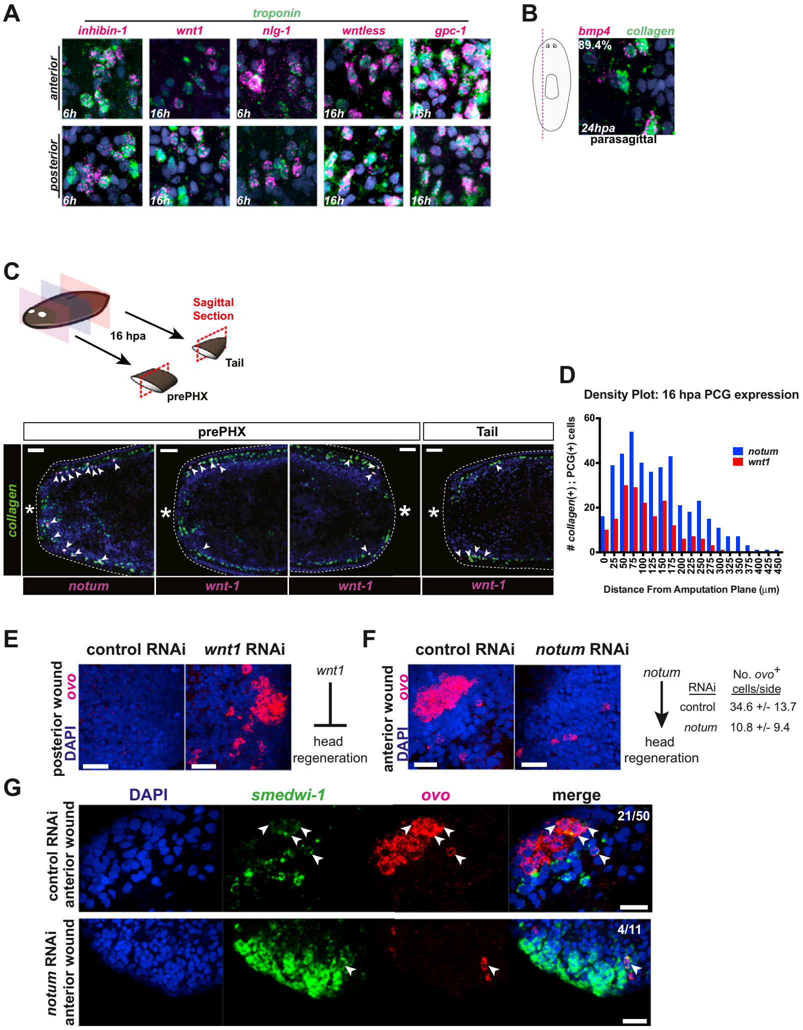

Figure S4. Expression and Role of PCGs in Regeneration, Related to Figure 4

(A) Animals (not irradiated) were examined at 6 or 16 hr after amputation as indicated, depending upon the peak time of wound-induced expression (21). Wound-induced genes were coexpressed together with *troponin*.

(B) *bmp4* expression at 24hpa in parasagittal fragments, as indicated in the schematic. Percentage overlap quantified from three unirradiated parasagittal animal fragments.

(C) Analysis of PCG and *collagen* coexpression in sagittal sections. Schematic illustrates surgical procedure. Transverse amputations were performed to produce fragments for analysis of wound-induced PCG expression: a tail fragment containing an anterior-facing wound site (see Figure 4), and a "pre-pharyngeal" (prePHX) fragment containing both anterior and posterior-facing wound sites. In tail fragments, 95.7% of *wnt1*-expressing cells also expressed *collagen* (n = 180/188) and 98.4% of *notum*-expressing cells also expressed *collagen* (n = 418/425). Tissue fragments were fixed 16 hr post-amputation, labeled by FISH and DAPI, and analyzed in sagittal sections. Zoomed images from 16 hpa pre-pharyngeal and tail fragments assessing PCG and *collagen* coexpression in multiple wound locations and orientations. Asterisks indicate approximate amputation site. Dotted lines indicate animal boundary. Scale bars, 50 µm.

(D) The distribution of *notum*$^{+}$ / *collagen*$^{+}$ and *wnt1*$^{+}$ / *collagen*$^{+}$ cells along the AP axis of tail fragments is shown in 25 µm bins.

(E–G) Defects in neoblast eye lineage-specification following *notum* or *wnt1* RNAi. *ovo* is expressed in eye progenitors during eye head regeneration, including in neoblasts. During regeneration, *wnt1* and *notum* were expressed in muscle cells at wounds and were required for formation of normal *ovo*+ neoblast numbers at wounds (normally none at posterior and numerous at anterior-facing wounds).

(E) 2/5 *wnt1(RNAi)* animals had *ovo*$^{+}$ clusters of cells at posterior-facing wounds 4 days following amputation (3 total clusters were present, and none were present in control RNAi (n = 4) animals). All three posterior *ovo*$^{+}$ cell clusters possessed multiple *smedwi-1*$^{+}$ / *ovo*$^{+}$ cells (see Figure 4E). Anterior, left.

(F) *notum(RNAi)* animals had too few *notum*$^{+}$ cells at anterior-facing wounds 48 hr following amputation (n $\geq$ 7 anterior blastema left or right sides were counted; shown are means ± SDs; p < 0.005, unpaired t test). Anterior, left.

(G) Optical sections of anterior-facing wounds show example *smedwi-1*$^{+}$ / *ovo*$^{+}$ cells (arrowheads). From sections spanning the entirety of the eyes shown (only sections are shown) 21/50 *ovo*$^{+}$ cells were also *smedwi-1*$^{+}$ in the control and 4/11 *ovo*$^{+}$ cells were also *smedwi-1*$^{+}$ in *notum* RNAi. Anterior, left for the control and down for *notum* RNAi. Bars, 20 µm.
